# Deep‐Learning‐Driven High‐Fidelity In Vivo Hyperspectral Fluorescence Imaging Under Extreme Photon‐Limited Conditions

**DOI:** 10.1002/advs.76802

**Published:** 2026-07-27

**Authors:** Renjian Li, Shutao Wu, Kaixiang Li, Zhenyu An, Yuwen Ben, Guiye LI, Sunil Kumar, James Mcginty, Tawfique Hasan, Songnian Fu, Meng Zhang, LingLing Chen

**Affiliations:** ^1^ College of Health Science and Environmental Engineering Shenzhen Technology University Shenzhen People's Republic of China; ^2^ Photonics Group Department of Physics Imperial College London London UK; ^3^ Advanced Electrical Engineering Division Engineering Department Cambridge University Cambridge UK; ^4^ Institute of Advanced Photonics Technology School of Information Engineering Guangdong University of Technology Guangzhou People's Republic of China; ^5^ Advanced Interdisciplinary Institute of Satellite Applications Beijing Normal University Beijing People's Republic of China

**Keywords:** deep learning, denoising, hyperspectral imaging, nano‐plastics

## Abstract

In vivo hyperspectral fluorescence imaging (fHSI) has transformed biomedical research by enabling qualitative/quantitative analysis of multiplexed molecular interactions. However, constraints for in vivo imaging and division of photons across numerous spectral channels create extreme photon‐limited conditions, where deep signal‐to‐noise coupling compromises fidelity and prevents accurate analysis. Here, we present a co‐designed confocal line‐scanning hyperspectral light‐sheet microscopy and dual‐stream residual attention network with non‐negative matrix factorization (DsRAN‐NMF), achieving high‐fidelity in vivo fHSI with up to three orders‐of‐magnitude improvement in photon efficiency. Advanced illumination and optical sectioning provide higher‐quality initial signals, restored by our DsRAN‐NMF with improved spatial and spectral fidelity, which simultaneously comprehends noise physics, high‐dimensional data geometry, and hyperspectral unmixing objectives to recover biologically interpretable spectral contributions. This approach resolves highly spectrally overlapping fluorophores at micron‐scale resolution in whole live zebrafish and enables visualization of nanoplastic uptake and circulation, establishing a pathway toward 4D hyperspectral imaging of living systems and nanoplastic toxicology.

## Graphical abstract

Highly overlapping fluorescent signals become distinguishable in photon‐limited living organisms via advanced imaging with intelligent reconstruction. The resulting in vivo hyperspectral imaging capability reveals nanoplastic uptake and circulation in live zebrafish, providing a new approach for studying complex biological and environmental processes.

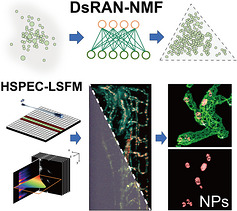

## 1 Introduction

Fluorescence microscopy is an indispensable imaging tool that can reveal structural and functional details of specimens, and has significantly contributed to new discoveries of various physiological mechanisms [1]. The rapid development of in vivo fluorescence imaging has enabled the direct investigation of complex biological processes within their native physiological context, and mesoscopic model organisms such as the zebrafish provide a powerful platform that bridges high‐resolution cellular dynamics with the systemic complexity of an intact vertebrate. To interpret these complex processes, multiple fluorescent labels are often used to simultaneously track different molecular species, facilitating the downstream analysis of their physical relationships and functional interactions [2, 3, 4]. In order to efficiently distinguish many such colors, hyperspectral fluorescence imaging (fHSI) has emerged as a powerful technique that captures the complete high‐resolution spectrum at each pixel of the whole field‐of‐view [5, 6, 7, 8]. This rich spectral information enables computational spectral unmixing to mathematically disentangle the individual contributions of spectrally overlapping fluorophores and background autofluorescence. In this way, the workflow is established as a quantitative methodology for precise and simultaneous analysis of numerous interacting components. Such capability is essential for unraveling the intricate mechanisms that govern living systems.

However, in vivo fHSI remains challenging, especially in mesoscopic organisms such as the zebrafish. This is due to the inevitable inhomogeneous scattering and absorption as well as pervasive endogenous autofluorescence within living tissue that collectively degrade spatial resolution and spectral fidelity. This challenge is critically compounded when monitoring dynamic processes, as a fundamental trade‐off exists between achieving an adequate signal‐to‐noise ratio (SNR) and maintaining viable experimental conditions, e.g., short exposure times for temporal dynamics, and bioacceptable experimental conditions including low excitation power and total light dose [9, 10]. These constraints inherently create an extremely photon‐budget‐limited situation, which is exacerbated by the fHSI process itself, as it divides the already scarce emission photons from each pixel across hundreds of spectral channels. Consequently, the exceptionally low per‐channel photon count renders the faint signal virtually inseparable from multi‐source complex noise, including sample‐dependent scattering, signal‐dependent photon shot noise, and random thermal noise. This deep signal‐noise coupling fundamentally compromises the fidelity of any subsequent quantitative analysis [11, 12].

In order to address this photon‐budget limited issues in such challenging circumstances, a number of sophisticated approaches in fHSI have been developed to improve its performance. In terms of optical instruments, light sheet microscopy and multiphoton microscopy have been coupled with fHSI to substantially reduce the scattering, phototoxicity, and photobleaching [13, 14, 15]. In terms of post‐processing methods, computational denoising approaches have been proposed to boost HSI SNR, including classical algorithms based on spatial and transform domain filtering/optimization [16, 17, 18] and deep‐learning‐based denoising methods relying on convolutional neural networks (CNNs), e.g., HSI‐DeNet [19], QRNN3D [20], DSTrans [21] and SSCDN [22]. These deep‐learning‐powered denoising approaches exhibit superior performance in remote sensing HSI applications, providing visually pleasing images. However, they have limited robustness, interpretability, and generalizability, and thus deviations from pixel‐wise alignment, reductions in training dataset size, and domain gaps across imaging modalities can substantially impair performance. Consequently, their application to in vivo mesoscopic organ‐scale fHSI is severely constrained by the poor generalization across heterogeneous specimens, the scarcity of training data, and the inherent difficulty of creating adequate realistic datasets. This challenge is particularly critical when samples undergo fast dynamics [23, 24]. A prime example of this data scarcity scenario is the investigation of nanoplastic (NP) movement or accumulation in zebrafish.

More importantly, the aforementioned deep coupling of multi‐source complex noise with authentic signal in in vivo mesoscopic fHSI culminates in a severe distortion and collapse of the high‐dimensional data manifold (Supplementary S1). While state‐of‐the‐art (SOTA) content‐aware networks have demonstrated remarkable success in improving metrics (e.g., peak signal‐to‐noise ratio (PSNR), structural similarity index (SSIM)), their restoration is highly likely to eradicate or distort the critical intrinsic spectral structure under such severe signal‐to‐noise coupling degradation scenarios due to their strong propensity to over‐smooth subtle yet important features [25, 26, 27]. The intrinsic pathological characteristics of initially degraded data persist following such restoration. Moreover, these models may even surreptitiously induce ill‐posedness via systematic bias (Supplementary S1). Such cascading limitations culminate in the complete failure of accurate downstream analysis through hyperspectral unmixing (Supplementary S1), which is essential for extracting authentic and useful information. Therefore, while in vivo fHSI offers the potential for quantitative and simultaneous analysis of numerous interacting components of physiological processes in their native environment, current methods fail to provide faithful information under extreme photon‐budget‐limited situations.

To address these limitations, we present an approach for high‐fidelity in vivo fHSI under extreme photon‐budget‐limited conditions. Our strategy is based on co‐development of advanced imaging systems providing higher‐quality initial signals, together with intelligent computational algorithms capable of extracting meaningful information from imperfect, photon‐budget‐limited data. To achieve this, we develop a confocal line‐scanning hyperspectral light sheet fluorescence microscope with the capability to mitigate scattering, minimize phototoxicity, and yield intrinsic optical sectioning. In addition, we propose a dual‐stream residual attention network combined with a non‐negative matrix factorization (DsRAN‐NMF) network with the capability of simultaneously comprehending the physics of noise, the geometry of the high‐dimensional data, and the objectives of the hyperspectral unmixing task, thereby resolving the existing paradox between HSI restoration and accurate hyperspectral unmixing analysis. We characterize DsRAN‐NMF and other SOTA networks and find that our DsRAN‐NMF reduces learning uncertainty, enhances data efficiency, and improves denoising effectiveness, and outperforms SOTA denoising methods in denoising and interpretable performance, especially in low‐data regimes (i.e., 6–13 pairs). Furthermore, we demonstrate that this approach successfully addresses the complex signal‐to‐noise‐deep‐coupling fHSI degradation, and verify the accuracy of downstream analysis in the resolving of spectrally largely overlapping targets. Finally, we demonstrate the in vivo applicability of the developed approach by combining whole‐fish 3D confocal‐HSPEC‐LSFM for time‐point volumetric mapping of NP biodistribution with local 2D hyperspectral imaging for dynamic tracking of NP movement in blood vessels, showing its potential as a practical tool for monitoring dynamic interactions in live biological samples.

## 2 Results and Discussion

### 2.1 Development of Confocal Line‐Scanning Hyperspectral Light Sheet Fluorescence Microscope

In vivo fluorescence spectral imaging under extreme photon‐limited conditions is challenged by coupled spatial and spectral information degradations during acquisition. In heterogeneous biological tissues, residual scattering and out‐of‐focus background fluorescence reduce raw spatial fidelity, even under light‐sheet illumination. At the same time, low per‐channel photon counts together with broad and spectrally overlapping emissions, particularly from tissue autofluorescence, compromise spectral separability and downstream unmixing reliability. These limitations are further increased in conventional multi‐channel imaging, where sparse spectral sampling may inadequately characterize complex mixed spectra under limited photon budgets.

**FIGURE 1:**
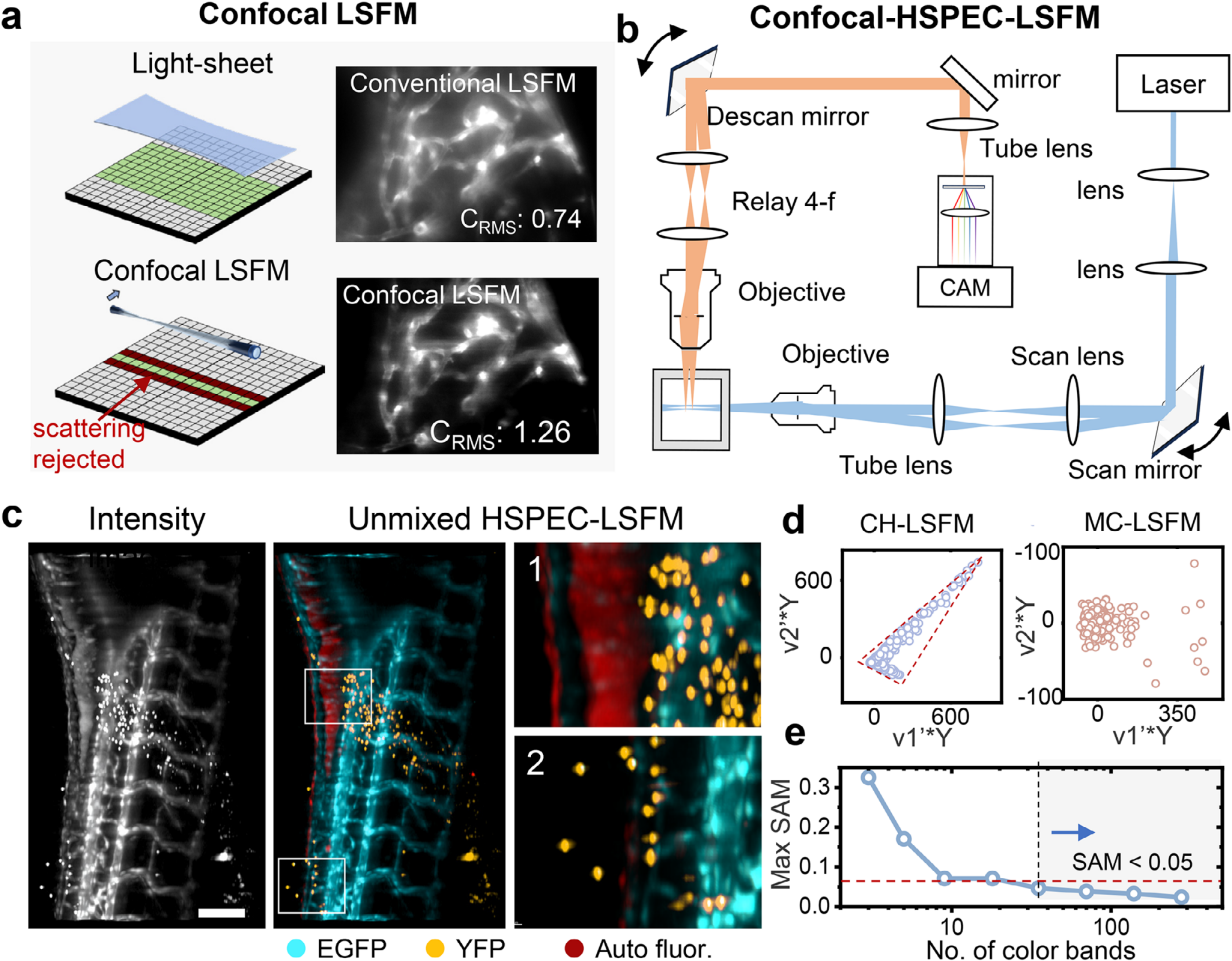
Confocal‐HSPEC‐LSFM improves raw spatial fidelity and enables hyperspectral component separation. (a) Confocal line‐scanning LSFM improves raw image contrast. (b) Optical configuration of Confocal‐HSPEC‐LSFM (details in Supplementary S4). (c) Hyperspectral unmixing of zebrafish vasculature, nanoplastics, and autofluorescence. (d) PCA comparison of hyperspectral and multi‐channel LSFM data. (e) Dependence of unmixing fidelity on spectral‐band number. CH‐LSFM: confocal‐HSPEC‐LSFM; MC‐LSFM: multi‐channel LSFM.

To mitigate these acquisition‐stage degradations prior to computational restoration, a confocal line‐scanning hyperspectral light‐sheet fluorescence microscope (confocal‐HSPEC‐LSFM) was developed. Compared with conventional LSFM, the synchronized line‐scanning and slit‐confocal detection configuration suppresses residual scattered and out‐of‐focus fluorescence that is not fully rejected by light‐sheet illumination alone, thereby improving raw spatial contrast at the acquisition stage (i.e., yielding an overall ∼2.3‐fold contrast improvement, Figure 1a and Supplementary S2). Hyperspectral detection was further integrated into the confocal LSFM optical path to acquire four‐dimensional spatial‐spectral data cubes (Figure 1b). Compared with conventional multi‐channel imaging, hyperspectral acquisition preserved richer spectral information for distinguishing spatially mixed and spectrally overlapping components in vivo, including EGFP‐labeled vasculature, YFP‐labeled nanoplastics, and broad abdominal autofluorescence backgrounds. Principal component analysis (PCA) of the acquired spectra revealed that conventional multi‐channel LSFM produced comparatively compressed and less separable spectral distributions, whereas hyperspectral acquisition retained improved spectral organization and separability in feature space, suggesting greater suitability for downstream spectral unmixing analysis (Figure 1d).

To further evaluate the contribution of spectral sampling density, channel‐number analysis was performed, and progressive degradation of endmember reconstruction accuracy was observed with increasingly sparse spectral sampling (Figure 1e, Supplementary S3). Consistent with the improved spectral separability observed in hyperspectral acquisition, NMF applied directly to the raw confocal‐HSPEC‐LSFM measurements recovered distinct abundance maps together with their corresponding spectral signatures for EGFP‐labeled vasculature, YFP‐labeled nanoplastics, and autofluorescence backgrounds (Figure 1c, Supplementary S3).

### 2.2 Development of DsRAN‐NMF Network

**FIGURE 2:**
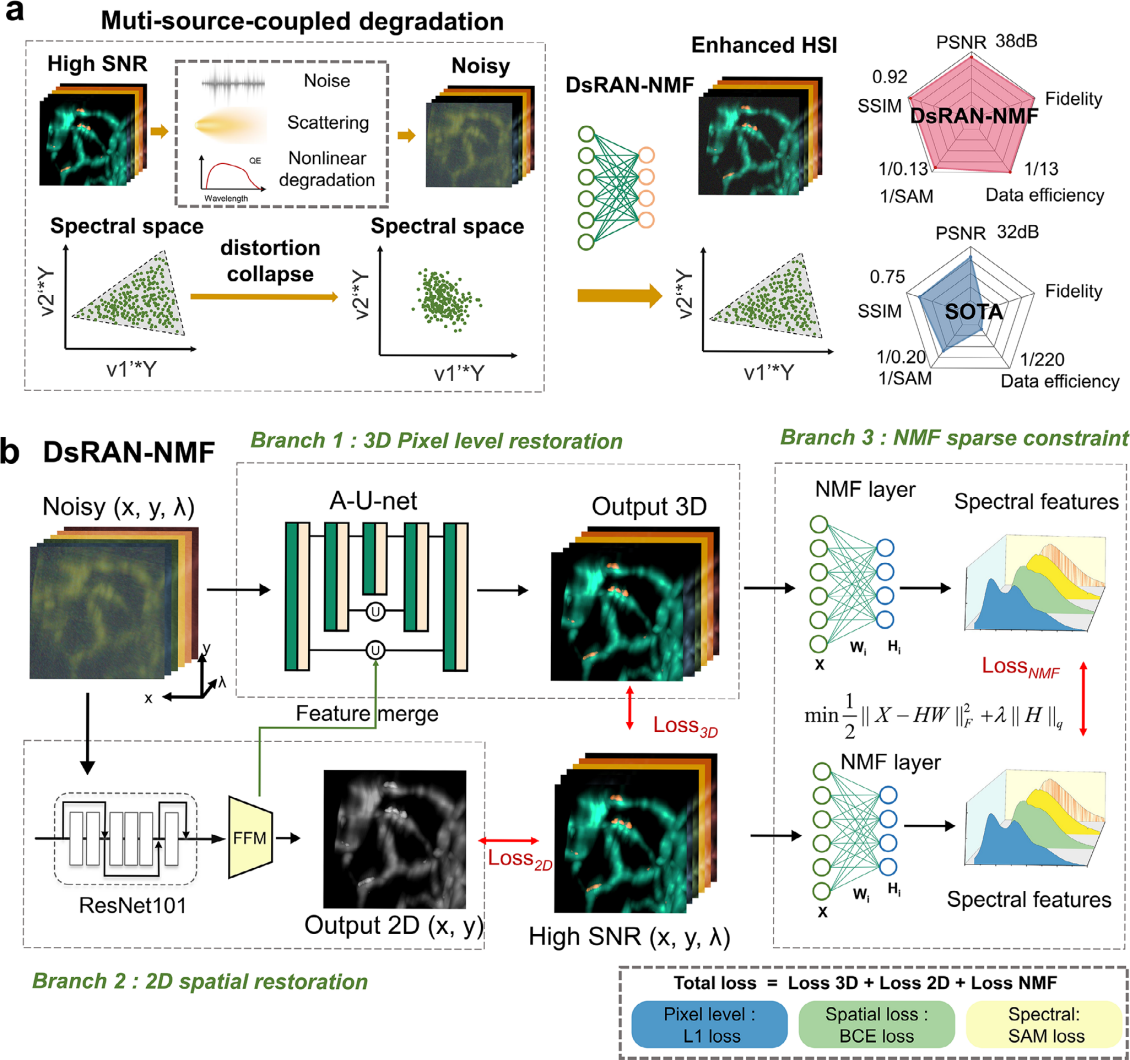
The architecture of the DsRAN‐NMF network during the training phase in the context of the hyperspectral light‐sheet fluorescence microscopy setup. (a) Spectral space collapse in HSI data from multi‐source nonlinear degradation, ill‐posed reconstruction by SOTA deep learning denoising methods, and high‐fidelity restoration by DsRAN‐NMF. (b) DsRAN‐NMF network employs a dual‐stream parallel architecture integrating ResNet‐101, Attention U‐Net with an unsupervised learning NMF providing sparse constraint as a spectral feature extraction layer. The total loss comprises three components: pixel‐level differences (L1 loss between the A‐U‐Net branch's 3D output and high‐SNR reference), spatial differences (BCE loss between the ResNet‐101 branch's 2D output and high‐SNR reference ’s intensity image), and low‐rank spectral differences (SAM loss comparing spectral features from NMF layers in the 3D output with high‐SNR reference). (c) Optical geometry of hyperspectral light sheet fluorescence microscopy system. SNR: signal‐to‐noise ratio. SOTA: state‐of‐the‐art. NMF: nonnegative matrix factorization. CAM: camera.

Building upon the acquisition‐stage improvements provided by confocal‐HSPEC‐LSFM, computational restoration remained necessary under extreme photon‐limited in vivo imaging conditions. In living systems, excitation intensity and exposure time are constrained by phototoxicity and photobleaching, while hyperspectral acquisition further partitions the limited photon budget across numerous wavelength channels, resulting in extremely low per‐channel photon counts even after optical scattering suppression. Under these conditions, computational restoration must suppress stochastic photon noise while preserving the spectral structure required for downstream hyperspectral unmixing (Supplementary S5). Conventional denoising methods often improve apparent image quality at the expense of subtle inter‐channel spectral relationships that are critical for reliable component discrimination. These coupled requirements motivated the development of DsRAN‐NMF, designed to jointly account for photon‐noise characteristics, hyperspectral spectral correlations, and downstream unmixing capability (Figure 2a).

The framework of the developed DsRAN‐NMF network is illustrated schematically in Figure 2b, employing a dual‐stream parallel architecture integrating ResNet‐101 [28] and Attention U‐Net [29] with an unsupervised learning NMF [30] that provides a sparse constraint as the spectral feature extraction layer. The dual‐stream framework not only leverages both spatial and spectral information from hyperspectral images, but also captures both global perception and local details. The first branch employs ResNet‐101 for spatial feature extraction, where 2D convolutions capture deep spatial information, followed by a Feature Fusion Module (FFM) that combines multi‐scale features and performs up‐sampling. The second branch employs Attention U‐Net, which integrates both spatial and spectral information through 3D convolutions and enhances signal‐critical features using a gating attention mechanism in the decoder. The decoder combines features from the encoder and the attention mechanism to restore spatial resolution while maintaining spectral details. These two branches work in tandem, with the first branch providing initial spatial features and the second branch refining them, leading to enhanced hyperspectral image reconstruction. Compared with monolithic networks, the developed DsRAN is more effective and comprehensive in preserving global perception and fine‐grained details through the synergy between ResNet101 and Attention U‐Net. Critically, it maintains performance across various SNR conditions and reduces oversmoothing/overfitting artifacts through these complementary streams, offering a framework for high‐fidelity restoration under non‐stationary noise scenarios.

Furthermore, to overcome the challenge of spectral composition sparsity, we employ NMF as the final spectral feature extraction layer to provide highly interpretable low‐rank representations for restoring spectral information. This enables the network to learn the mapping from spatial superposition spectra to authentic spatial components rather than the simplified pixel‐to‐pixel spectral reconstruction used in conventional deep learning approaches. This strategy, which utilizes unsupervised learning to guide the learning process through sparse constraints, can provide more fundamental spectral signature information and more critical features, reducing the risk of overfitting and enhancing generalizability, especially when the training dataset is small. During training, a multi‐loss‐joint strategy is adopted for DsRAN‐NMF to balance the spatial and spectral restoration. The total loss is set as the sum of pixel‐level discrepancy (represented by *L*_1_ [31]), 2D spatial loss (represented by binary cross‐entropy loss (*L*_BCE_) [32]), and 1D spectral loss (represented by spectral angle mapper (SAM) [33]). Further architecture details and explanations of DsRAN‐NMF can be found in Supplementary S6.

### 2.3 Denoising Performance Benchmarking Using Large‐Quantity and High‐Quality Training Data

**FIGURE 3:**
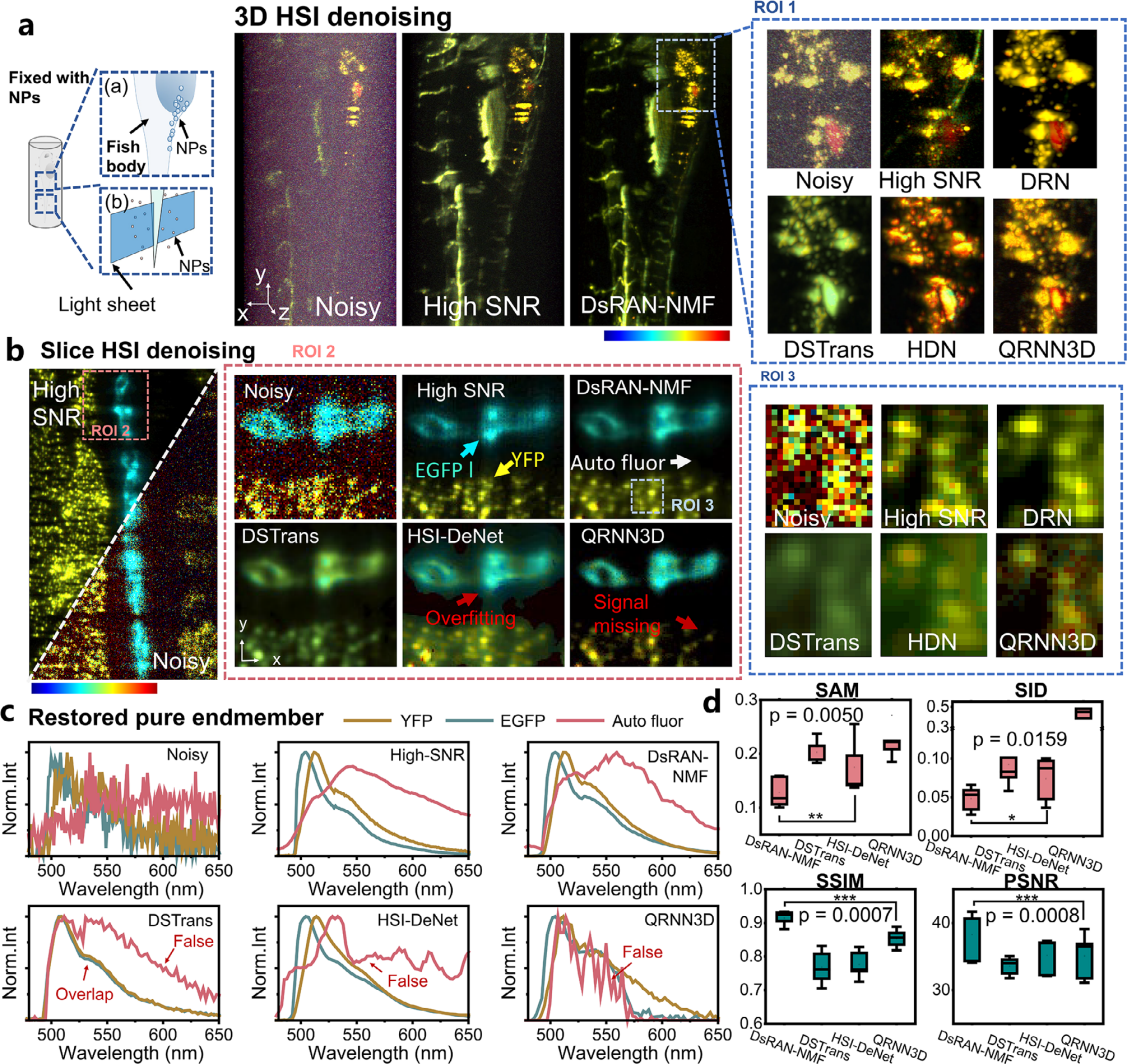
Denoising performances benchmarking using DsRAN‐NMF and representative SOTA models with high quantity and quality training datasets. (a) 3D volume visualizations via max intensity projection (MIP) of zebrafish abdomen acquired with low‐SNR (Noisy), high‐SNR hyperspectral light sheet fluorescence microscope images and the denoised results using DsRAN‐NMF, DSTrans, HSI‐DeNet and QRNN3D, in which color represents the peak wavelength. ROI 1 (blue rectangle): nanoplastics in the gastrointestinal tract of zebrafish. Color bar: 500–520 nm. (b) 2D slice images of zebrafish tail with nanoplastics in x‐y plane and zoomed‐in images in the ROI 2 (red rectangle) and ROI 3 (blue rectangle). Color bar: 490–525 nm. (c) Typical spectra (pure endmembers) of Noisy, high‐SNR reference and denoised images in (b). (d) SAM, SID, SSIM, and PSNR calculated from the restored slice images in (b), n = 5 patches from (b). DRN: DsRAN‐NMF; HDN: HSI‐DeNet.

To quantitatively evaluate DsRAN‐NMF denoising performance, we applied it to zebrafish and fluorescent nanoplastics under HSPEC‐LSFM for 3D mesoscopic imaging. A total of 220 paired images of transgenic zebrafish (Tg (kdrl: EGFP) embedded in 1.5% w/w agarose containing fluorescent NPs (diameter of 200 nm, YFP with excitation/emission at 505/515 nm) including low‐SNR hyperspectral images (with low exposure time of 30 ms and low illumination power of 35 µW) and high‐SNR images, were acquired as input and high‐SNR datasets respectively, and all pairs were registered and screened at the pixel level before training and evaluation (details in Supplementary S7). The corresponding number of patches, independent slices/stacks, biological samples, and split protocol are reported in Supplementary S8. Figure 3a shows the experimentally obtained hyperspectral fluorescence images and the corresponding denoised images using DsRAN‐NMF and representative SOTA models (i.e., DSTrans [21], HSI‐DeNet [19], and QRNN3D [20]). Compared with SOTA networks, our DsRAN‐NMF significantly suppresses noise and restores information with high‐fidelity in both spectral and spatial domains (Figure 3a–c), and clearly exhibits higher statistical accuracy in terms of SAM (∼26.8%–42.4% improvement), spectral information divergence (SID [34], ∼34.9%–89.3% improvement), structural similarity index (SSIM [35], ∼7.2%–19.9% improvement) and peak signal‐to‐noise ratio (PSNR, ∼3.12–4.7 dB improvement) between the restored hyperspectral images and high quality high‐SNR (Figure 3d).

As features propagate through these networks, the constrained receptive field, limited stacking depth, and the inferior capability to handle the disruptive influence of excessive multiplicative noise render HSI‐DeNet and QRNN3D incapable of sufficient identification of complex structures characterized by significantly varying signals in hyperspectral fluorescence images (Figure 3b,c). Even with the inclusion of a dual‐stream attention Transformer block to enhance the capture of long‐range dependencies, DSTrans still struggles with fine‐grained detail preservation (Figure 3b ROI 3). The results of DSTrans, QRNN3D, and HSI‐DeNet exhibit oversmoothing, overfitting, and loss of spatial structures (Figure 3a,b), as well as erroneous, even completely mistaken pure endmember spectral signatures (Figure 3c), both of which are manifestations of model biases. These shortcomings are significantly alleviated by DsRAN‐NMF, thus more fine structures and intricate details such as NP morphology, YFP and EGFP spectra, and low‐signal autofluorescence can be restored accurately. Succinctly, the synergy between ResNet‐101 and Attention U‐Net, along with utilizing NMF as the final spectral feature extraction layer, endows DsRAN‐NMF with the ability to effectively and comprehensively account for the physics of noise, the geometry of the spatio‐spectral high‐dimensional data, ultimately addressing the complex pathological characteristics of data under signal‐to‐noise‐deep‐coupling degradation.

### 2.4 DsRAN‐NMF Supports Downstream Analysis with Hyperspectral Unmixing in in vivo Imaging

**FIGURE 4:**
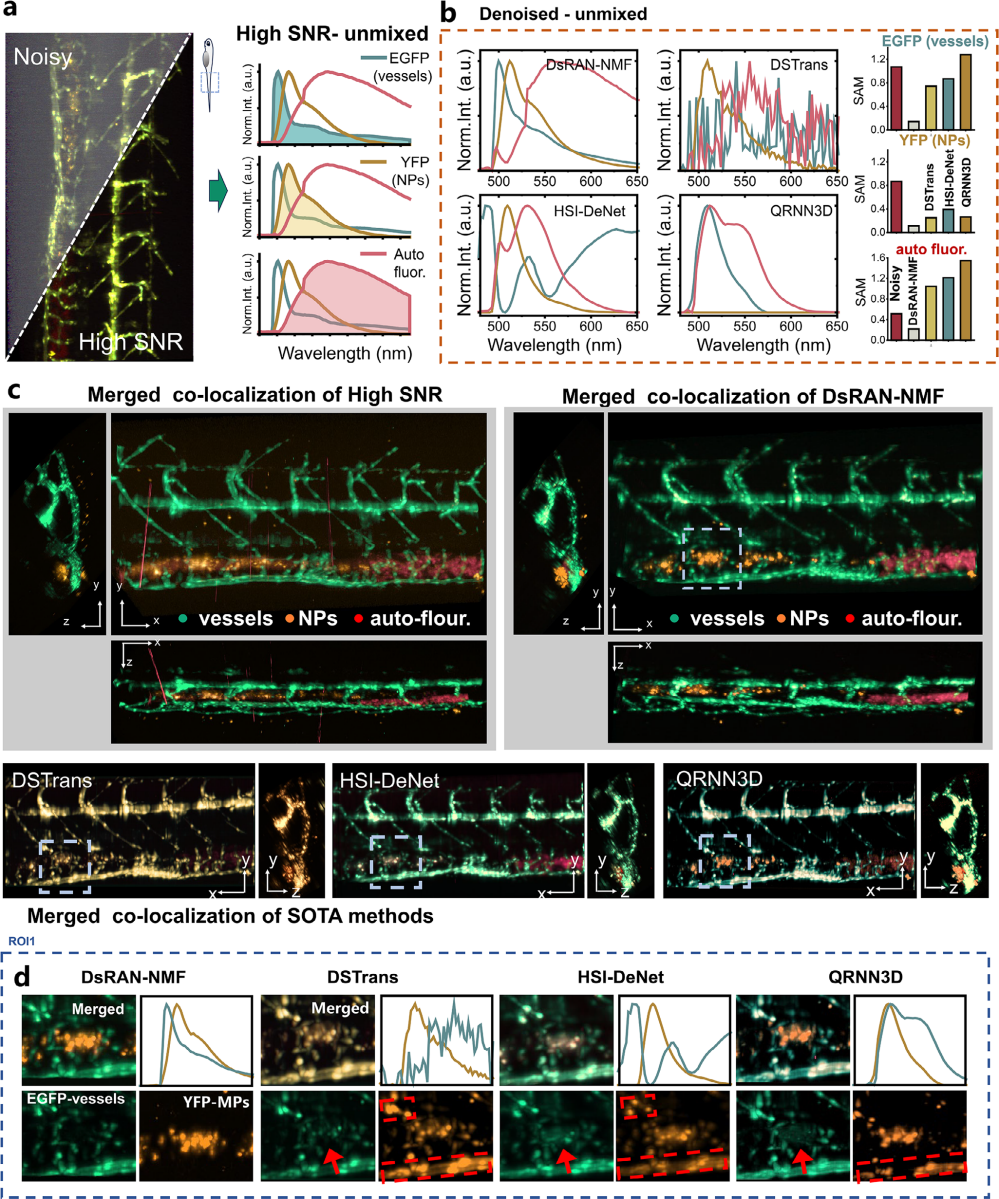
Unmixing results of denoised 3D hyperspectral fluorescence images. (a) Noisy and high‐SNR hyperspectral fluorescence images and the endmember spectra from high‐SNR references unmixing. (b) The decomposed endmember information from linear unmixing on the restored results of DsRAN‐NMF, DSTrans, HSI‐DeNet, and QRNN3D, and the SAM between these results with the unmixing results from the high‐SNR references. (c) The merged co‐localization results of high‐SNR and denoised hyperspectral fluorescence and (d) the zoomed‐in area of ROI (white rectangle), in which red arrows and red rectangles indicate misidentification of target signals in SOTA denoising results. The different color of the images represents the corresponding endmember of unmixing results from hyperspectral fluorescence images (i.e., Green: vessels, Orange: NPs, red: auto‐fluorescence). The initialization robustness and rank‐selection analysis of NMF unmixing are shown in Supplementary S10.

More importantly, the denoised results are expected to enable accurate analysis of physical relationships and interactions among selected targets. Considering the fluorophores with relatively large overlap (e.g., EGFP and YFP) or broadband autofluorescence, hyperspectral unmixing is commonly employed for interpreting hyperspectral data, where each pixel may contain spectral signature contributions from multiple constituent endmembers [36, 37]. To evaluate the performance of DsRAN‐NMF in a more complex application scenario where accurate endmember identification and quantification are essential, we applied it to live transgenic zebrafish embryos (Tg (kdrl: EGFP) uptaking NPs (200 nm fluorescent polystyrene beads with peak excitation/emission at 505/515 nm) using the hyperspectral light sheet fluorescence microscope system. Subsequently, linear non‐negative spectral unmixing was performed as a practical approximation to estimate the endmember spectra and abundance maps of the three dominant fluorescent contributors: EGFP‐labeled endothelial cells and vascular structures, YFP‐labeled NPs, and weak intrinsic intestinal autofluorescence (details of NMF in Supplementary S9 and S10). Figure 4a shows the noisy and high‐SNR hyperspectral fluorescence images and the endmember information from high‐SNR reference unmixing. The restoration model used here was trained on the fixed/cleared paired dataset described above and directly applied to these live zebrafish fHSI data, with cross‐state validation summarized in Supplementary S11.

Figure 4b shows the decomposed endmember information from linear unmixing on the restored results of DsRAN‐NMF, DSTrans, HSI‐DeNet and QRNN3D, exhibiting the superior performance of DsRAN‐NMF with a high correspondence to the actual components (i.e., EGFP, YFP and autofluorescence). Subsequently, the merged reconstructed co‐localization results of separate structures were obtained to illustrate the physical relationships (Figure 4c,d). DsRAN‐NMF demonstrates an effective restoration for accurate analysis of overlapping structures after linear unmixing with the superior determination of their spectra from low‐SNR data sets of multiple labels and autofluorescence. The relatively faint autofluorescence signal is also successfully determined and separated, providing additional information of morphological context. In contrast, the images processed by DSTrans, HSI‐DeNet, and QRNN3D not only exhibit a significant loss of weaker signals from autofluorescence, but also, more importantly, fail to determine the signature spectra from different labels and their corresponding spatial distributions. These results suggest that while these SOTA deep learning denoising methods provide seemingly acceptable denoising performance in terms of common evaluation metrics (e.g. SAM, PSNR, SSIM) and visually pleasing images (Figure 3a,b), their heavy focus on pixel‐level‐similarity content awareness is prone to over‐smoothing superficial recovery which lead to misidentification of key biomarkers, erroneous quantification of target signals, and overall diminished reliability in hyperspectral image analysis (Figure 4b,d)). Consequently, the adverse effects further degrade sensitivity and specificity for detecting environmental, pathological, and dynamic changes, ultimately impeding effective decision‐making and research outcomes. A considerable benefit of the developed DsRAN‐NMF is the clarity of highly interpretable low‐rank representations in the final spectral feature extraction layer, unlike that in hidden layers in traditional CNNs. With more fundamental spectral features extracted and used as physics‐based prior constraints, meaningful compact representations flowing through the model improve the interpretability of the learning process, supporting more reliable downstream analysis in hyperspectral fluorescence imaging applications. The developed framework addresses the challenge of transcending the separate domains of “denoising” and “unmixing”, preventing geometric collapse under signal‐to‐noise‐deep‐coupling degradation.

### 2.5 High‐Performance Learning of DsRAN‐NMF in Low‐Data Regime

As demonstrated before, DsRAN‐NMF achieves superior denoising performance and facilitates accurate hyperspectral image unmixing analysis on large‐quantity high‐quality training datasets. Meanwhile, data efficiency is crucial in HSI denoising especially for biomedical imaging of dynamic processes. This presents several challenges, including obtaining precise high‐SNR reference, limited availability of extensive training data, and significant variability in noise profiles. The requirement for large‐quantity and high‐quality datasets for conventional deep‐learning‐based hyperspectral denoising methods is significantly reduced by our DsRAN‐NMF. On one hand, the effective synergy of ResNet‐101 and Attention U‐Net leverages their complementary strengths, not only by improving high‐fidelity restoration and robustness against noise but also by lowering the risk of overfitting when the training size is reduced. On the other hand, utilizing NMF as the final spectral extraction layer to incorporate a sparse constraint enables dynamic bias adaptation. This flexibly adjusts model biases according to specific contextual scenarios, yielding highly interpretable, compact fundamental representations of the underlying data structure, guiding the learning process and improving data efficiency and stabilizing restoration under limited paired training samples.

**FIGURE 5:**
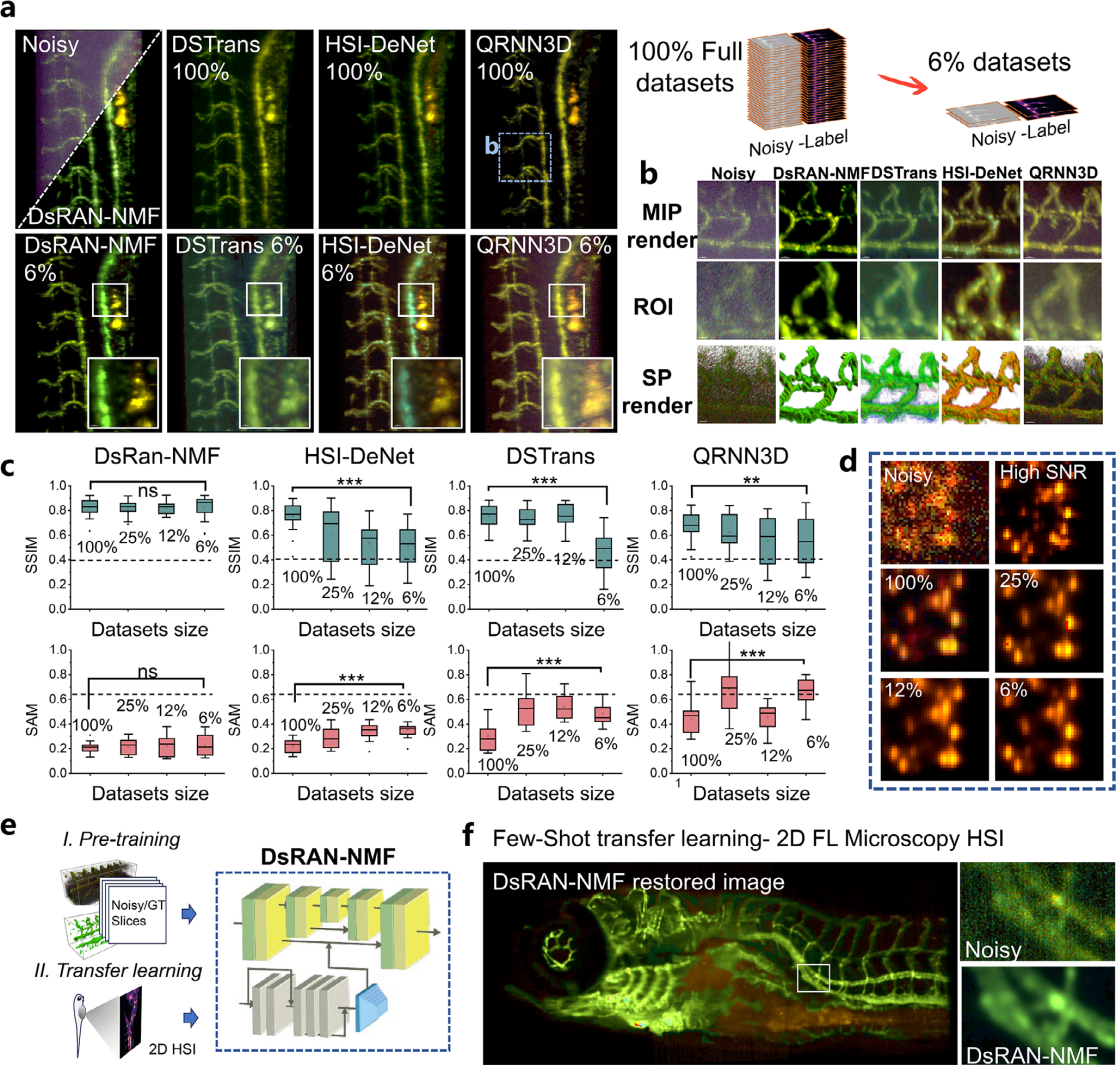
Denoising performance in low‐data‐learning on 2D and 3D hyperspectral fluorescence microscopy images. (a) 3D visualizations via MIP rendering of zebrafish denoised using learning‐based methods with full‐data learning and low‐data learning (i.e., 6% datasets, 13 pairs) using DsRAN‐NMF network and other SOTA methods, respectively. (b) zoomed‐in images of the ROI (blue rectangle in a) via MIP render and shadow projection (SP) render. (c) The variations of SSIM and SAM metrics of different learning‐based methods by gradually decreasing the training data size (i.e., 25%, 12%, 6%). Each boxplot was calculated at the independent slice/stack level after aggregating patch‐level measurements from the same slice/stack. Statistical comparisons were performed using two‐sided paired tests between the full‐data model and reduced‐data models for each method. ns, not significant; ^*^ *p* < 0.05; ^**^ *p* < 0.01; ^***^ *p* < 0.001. (d) Restored slice hyperspectral image of Nanoplastics using DsRAN‐NMF pre‐trained with 100%, 25%, 12% and 6% datasets, respectively. (e) Sketch map of transfer learning on 2D line‐scanning hyperspectral fluorescence images. (f) 2D hyperspectral image before and after restoration using DsRAN‐NMF with few‐shot transfer learning (6 pairs). Color bar: 500–520 nm in (d) and 490–525 nm in (a) and (f).

To verify the data efficiency of DsRAN‐NMF in low‐data regimes, Figure 5 compares the denoising performance and the slopes of SSIM and SAM metrics of different learning‐based methods as the training data size is gradually decreased (25%, 12%, 6%). The reduced subsets were constructed as representative low‐data training sets, and their biological sample composition, independent slice/stack numbers, and split protocols are summarized in Table S2. As the data size shrinks, the denoising quality of the traditional deep learning methods (i.e., DSTrans, HSI‐DeNet, and QRNN3D) declines sharply, e.g., SSIM decreased to ∼0.5, SAM increased to ∼0.6 (Figure 5c), and their substantial overfitting adversely effects fine‐grained details either by over‐smoothing or the loss of critical textures. In contrast, DsRAN‐NMF showed substantially reduced sensitivity to training‐data reduction. Under the representative 6% low‐data setting (details and stable performance down to 6–13 training pairs in Supplementary S8 and S26), DsRAN‐NMF maintained comparable SSIM and SAM performance to the full‐data model on the same independent test set owing to NMF‐guided learning and preservation of the compact low‐dimensional spectral manifold. This result suggests that the NMF‐guided spectral constraint reduces the dependence on large paired datasets by stabilizing the restoration of low‐dimensional spectral structure. Our unsupervised learning providing self‐adapted physics‐based prior sparse‐constraint dynamic biases helps the network to learn the denoising process more efficiently and effectively. Overall, DsRAN‐NMF not only reduces the dependency on extensive high‐quality datasets but also improves the model's ability to adapt to the unique sample‐imaging‐codependent noise characteristics and spectral properties of hyperspectral images, which are essential in data‐constrained in vivo biomedical imaging scenarios.

Furthermore, the transferability of the pre‐trained DsRAN‐NMF was evaluated on a related 2D line‐scanning hyperspectral fluorescence imaging modality in addition to the 3D light‐sheet fHSI data used in the pretraining stage. With the help of transfer learning using 6 additional images from 2 biological samples, the prediction accuracies are substantially enhanced (Figure 5e,f, Supplementary S12). Compared with other models on the same training data, our DsRAN‐NMF is able to produce robust predictive results, thereby facilitating broader applicability for data‐constrained in vivo biomedical imaging scenarios. These improvements in clarity and contrast exhibit enhanced photon efficiency by up to three orders of magnitude for fHSI (Supplementary S13), and benefit precise structure segmentation and statistical analysis, enabling more detailed observation of dynamic processes in vivo.

### 2.6 High‐Fidelity In Vivo fHSI Reveals Accumulation and Transfer of NPs in Zebrafish

Plastic pollution is prevalent in various environments, and fragmentation breaks plastic debris down to micro‐ and nano‐plastics [38, 39]. These micro‐ and nano‐plastics pose considerable risks to both ecosystems and human health. This is particularly the case for the nanoplastics (NPs) due to their distinct physicochemical characteristics such as high surface‐to‐volume ratios for contaminant adsorption and small size for easy penetration through biological barriers [40, 41]. Visualizing the uptake, distribution, and vascular‐associated accumulation of NPs in live zebrafish is important for understanding their in vivo transport behavior and for generating hypotheses for subsequent toxicological studies. Yet SOTA in vivo studies remain limited due to analytical difficulties, particularly under experimental conditions that require low illumination power and short exposure times to ensure bio‐validity and suppress motion blur [42]. Our high‐fidelity fHSI approach provides an opportunity for in vivo study of accumulation and transfer of NPs in zebrafish (experiment details and elaboration in Methods and Supplementary S14–S16, S20 and S22).

**FIGURE 6:**
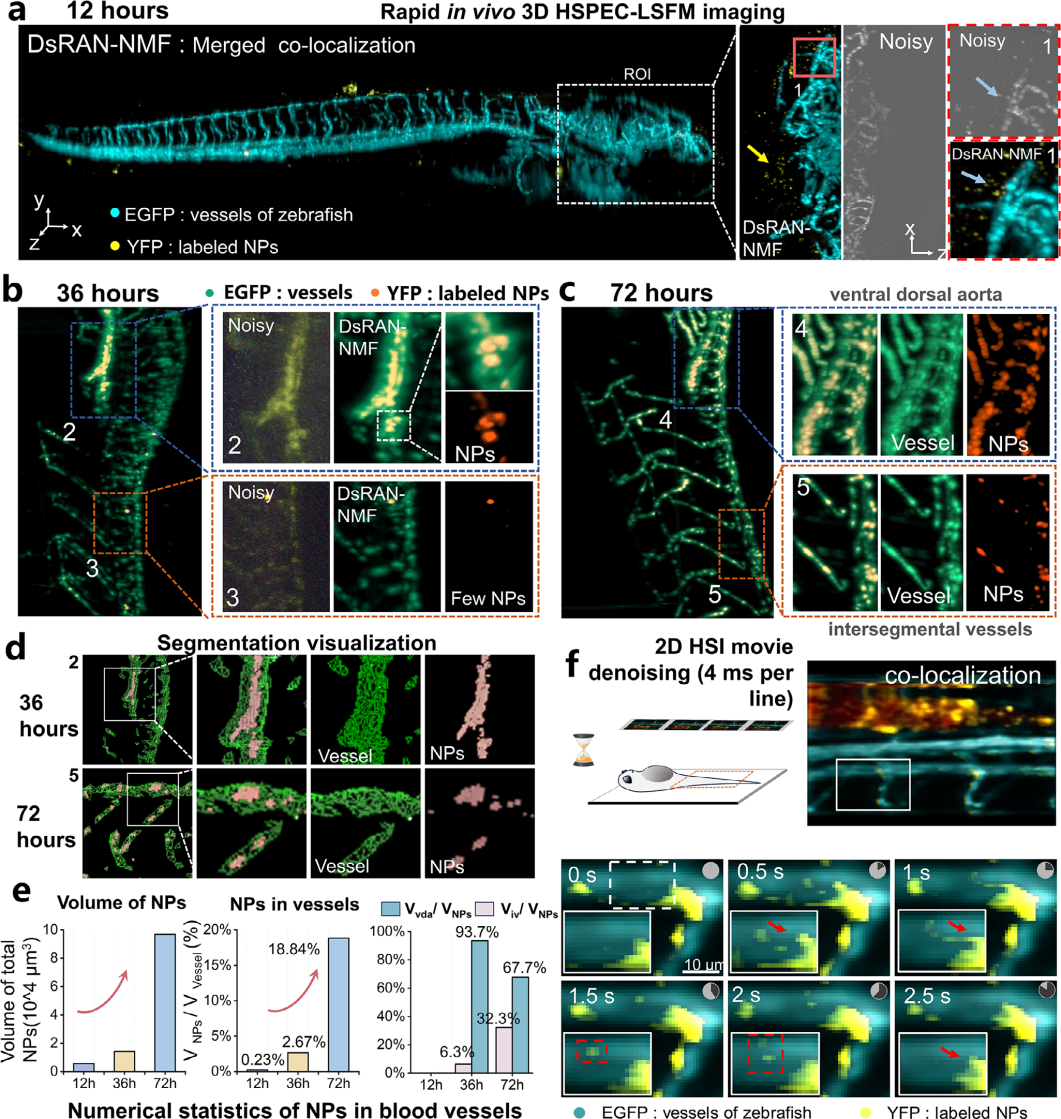
3D visualizations via MIP rendering and the corresponding segmentation results of zebrafish embryo enhanced by the low‐data pre‐trained DsRAN‐NMF network at multiple sampling time points (i.e., 12, 36, 72 h) after zebrafish exposure for nanoplastics uptake experiments and local 2D dynamic tracking of nanoplastics in live zebrafish. (a) Unmixed whole‐body 3D image of zebrafish and top view image of the fish head after 12 h of nanoplastics exposure. (blue: vessels, yellow: NPs). (b) Unmixed 3D image of zebrafish and zoomed‐in image of ROI (white rectangle) at 36 h after nanoplastics exposure. (green: vessels, orange: NPs). (c) Unmixed 3D image of zebrafish and zoomed‐in image of ROI (white rectangle) at 72 h after nanoplastics exposure. (d) Segmentation visualization of ROI 3 (from (b)) and ROI 4 (from (c)). (e) Numerical statistics of NPs in blood vessels. (f) Dynamic process images of nanoplastics accumulation in zebrafish vessels acquired by 2D light‐scanning hyperspectral fluorescence microscopy with DsRAN‐NMF enhancement, showing local NP accumulation when labeled particles moved from the main vessel toward secondary vessels.

Figure 6a–d show 3D visualizations via maximum intensity projection (MIP) rendering and the corresponding segmentation results of zebrafish embryo imaging enhanced by the low‐data pre‐trained DsRAN‐NMF network at multiple sampling time points (i.e., 12, 36, 72 h) after zebrafish exposure for NP uptake experiments. This demonstrates comparable improvements in image quality compared to the validation set, including improved visibility of structural details as well as the subsequent unmixing analysis (e.g., the cerebral blood vessels as shown in Figure 6a). The live zebrafish results are consistent with the cross‐state validation described above, in which the fixed‐trained model showed restoration performance comparable to a live‐trained model on live test data (Supplementary S11). The restored images therefore provide improved SNR and clearer mapping of vascular structures and labeled NP signals for subsequent unmixing and segmentation analysis.

3D volume imaging at multiple sampling time points (i.e., 12, 36, 72 h) after exposure shows the time‐lapse of NP accumulation and transfer process. These time‐point maps showed NP‐associated fluorescence initially concentrated in the intestinal region and later detected in vascular regions, including distal and secondary vessels (Figure 6a–d). The volumetric occupancy ratio of circulating‐accumulated NPs within zebrafish vasculature exhibits a ∼7‐fold increase, while the ratio of the volume of NPs in intersegmental vessels exhibited a ∼5‐fold increase between 36 and 72 h post‐exposure (Figure 6e). This suggests that small NPs may be more easily internalized via gills and intestines into the circulation, and then could be carried to any organ/tissue, especially the distal vessels (Supplementary S17). This agrees well with the previous observation that small‐sized NPs are prone to accumulate in internal organs [43].

To further illustrate the capability of our 2D DsRAN‐NMF‐enhanced line‐scanning hyperspectral fluorescence microscopy, we study the dynamic accumulation of circulating NPs by tracking high‐speed movement of NPs in the blood vessels (Figure 6f). The time‐lapse data can identify the trajectory of NPs, which is beneficial for revealing the underlying complex movement dynamics. While the typical blood flow in zebrafish caudal vessels ranges from 0.20 to 0.71 mm/s, the measured NPs speed is slower, especially with aggregation. A local vessel‐associated NP accumulation event was observed when labeled NPs moved from the main vessel toward secondary vessels (Figure 6f). These observations suggest that NPs can transfer within the vascular system and dynamically accumulate after exposure and uptake in live zebrafish, with potential to interfere with local vessel patency, particularly in smaller vessels where blood flow may be more vulnerable to particle accumulation. Such vessel‐associated NP accumulation may provide a relevant imaging clue for understanding plastic‐related vascular effects. Nevertheless, the underlying mechanism remains to be further clarified, and additional studies combining hemodynamic measurements, endothelial‐injury assessment, and micro‐/nanoplastics with different properties and sizes across animal models will be needed to more comprehensively evaluate the health risks posed by these emerging pollutants.

## 3 Conclusion

Our results demonstrate that the integration of confocal line‐scanning hyperspectral light sheet fluorescence microscope and the DsRAN‐NMF network provides in vivo high‐fidelity mesoscopic fHSI under extreme photon‐budget‐limited situations, mitigating the traditional trade‐offs between imaging speed, resolution, phototoxicity, and analytical fidelity. The advanced illumination and intrinsic optical sectioning jointly enhance the fidelity of the initially acquired data while minimizing phototoxicity, supporting repeated or time‐point live imaging under reduced excitation dose. The DsRAN‐NMF network leverages this improved signal, providing data‐efficient, highly interpretable restoration with cross‐state transferability, enabling hyperspectral unmixing to uncover meaningful authentic spectral features.

A primary limitation of the present work arises from the linear mixing assumption underlying the NMF formulation. Although linear spectral mixing remains the predominant model in fluorescence spectral imaging [36, 44] and is supported by the confocal line‐scanning acquisition employed in this work (elaborated details in Supplementary S3), it represents a simplified description of light‐tissue interactions. In highly heterogeneous biological environments, effects such as multiple scattering, fluorescence reabsorption, or other nonlinear optical interactions may introduce nonlinear spectral interactions that are not captured by the linear mixing model, potentially leading to biased abundance estimates or incomplete separation of spectrally mixed components. Future studies may benefit from incorporating nonlinear spectral mixing models or physics‐informed unmixing strategies [37, 45] to better account for these effects in complex in vivo settings.

Beyond the assumptions of the spectral unmixing model, the deep‐learning‐based restoration stage also introduces important considerations. Although the proposed network substantially improves reconstruction quality under photon‐limited conditions, like other deep‐learning‐based inverse models, it remains susceptible to reconstruction bias and potential artifact generation under domain shifts between training and deployment conditions. Such domain shifts may arise from tissue‐ or organ‐specific biological heterogeneity, developmental or physiological‐state variability, background autofluorescence, fluorophore composition, imaging depth, or instrument characteristics, potentially reducing restoration fidelity and affecting downstream analysis. In addition, the current method provides deterministic reconstructions without explicit uncertainty estimation and may become less reliable in regimes where the acquired measurements contain insufficient information for faithful recovery. Future developments may benefit from uncertainty‐aware restoration strategies, such as Bayesian deep learning, posterior sampling, or probabilistic inference frameworks, which could provide confidence estimates together with reconstructed hyperspectral data and help identify regions where restoration is less reliable [46, 47, 48, 49].

Despite these limitations, DsRAN‐NMF can be applied to additional biomedical samples and other hyperspectral datasets (details in Supplementary S18 and S19), suggesting its potential adaptability beyond the specific zebrafish‐nanoplastic imaging task. Future work should further evaluate this adaptability across broader biological specimens, imaging systems, and degradation regimes with standardized validation protocols. By unifying advanced imaging with intelligent reconstruction, our approach opens a new direction for long‐term, qualitative/quantitative, 4D spectral imaging of living systems and marks a step change in how spatiotemporal dynamics and interactions can be investigated under photon‐limited regimes.

## 4 Materials and Methods

### 4.1 Hyperspectral Fluorescence Imaging Setup

Hyperspectral light sheet fluorescence microscopy enables high‐throughput 3D spatial‐spectral mapping by integrating a wavelength‐resolved detection system with digital light sheet microscopy through the synchronization of line‐scanning illumination and de‐scanning detection, as shown in Figure 1b and Supplementary S2. In the illumination path, a scanning galvanometer (Thorlabs, GVS212) was employed to direct the 488 nm laser (Cobolt, Skyra) for line excitation of the field of view (FOV). The laser beam was reshaped by a set of lenses (Thorlabs, GAS0121; Thorlabs, TTL100‐A; Olympus, LMPlanFLN, 10x/0.25) to meet the light sheet illumination requirements. In the detection path, a 4‐f system comprising an objective lens (Olympus, UPlanFLN, 10x/0.3) and a tube lens (Thorlabs, TTL100‐A), along with a relay imaging system consisting of two achromatic lenses (Thorlabs, TTL100‐A; Thorlabs, AC508‐180‐A‐ML), was employed for magnified imaging. Another scanning galvanometer (Thorlabs, GVS212) was positioned at the front focal plane of the tube lens to enable synchronous de‐scanning, ensuring efficient coupling of the illumination line into the spectrograph (Princeton Instruments, Fergie), thereby enabling the acquisition of spectra corresponding to each voxel via grating spectroscopy. The in‐house developed hyperspectral light sheet fluorescence microscope system provides a 2.77 ± 0.29 µm/ 4.84 ± 0.59 µm resolution in the lateral /axial directions. All system controls and acquisition settings are implemented by a self‐developed LabVIEW program. With this advanced imaging technique, the developed fHSI system is capable of acquiring spectrally resolved optically‐sectioned data sets for large developing samples (a few hundreds of µm to mm) while maintaining the high spatial and temporal resolution (∼µm and ∼s/plane) under photon‐limited acquisition conditions. More details of HSPEC‐LSFM can be found in Supplementary S3 and S4.

### 4.2 Model

The dual‐stream residual attention network combined non‐negative matrix factorization (DsRAN‐NMF) network employs a two‐stream parallel architecture, DsRAN with an NMF spectral feature extraction layer, which effectively integrates the ResNet‐101 [28], Attention U‐Net networks [29], and unsupervised methods NMF [30], leveraging both spatial and spectral information from hyperspectral images, as it shows in Figure 2b and Supplementary S4.

The first branch leverages ResNet‐101 for spatial feature extraction, where 2D convolutions are used to capture deep spatial information, followed by a Feature Fusion Module (FFM) that combines multi‐scale features and performs up‐sampling. The second branch utilizes Attention U‐Net, which integrates both spatial and spectral information through 3D convolutions and enhances important features using a gating attention mechanism in the decoder. The decoder combines features from the encoder and the attention mechanism to restore spatial resolution while maintaining spectral details. These two branches work in tandem, with the first branch providing initial spatial features and the second branch refining them, leading to enhanced hyperspectral image reconstruction. More architecture details of DsRAN can be found in Supplementary S4.

An NMF layer was employed as the final spectral feature extraction layer to ensure the authenticity of restored image spectra. Specifically, the restored output image from DsRAN and the paired high‐SNR image was separately input into the NMF layer to obtain dimensionality‐reduced spectral features, of which the angular distance was calculated to measure the spectral restoration accuracy. NMF is commonly used for spectral image unmixing, which decomposes spectral images into non‐negative bases (spectra) and the product of their abundances. The decomposed spectra can usually map components with real physical meaning, which distinguishes NMF layers from hidden‐layer‐based spectral feature extraction. The use of NMF as a spectral feature extraction method enables the network to learn the mapping from spatial spectra to real spatial components during the learning process, rather than simply pixel‐to‐pixel style spectral recovery. Here, the NMF layer can provide more fundamental spectral structured information, improve data sparsity, reduce the risk of overfitting, and especially enhance the network's generalization ability when the training set is small. More details of NMF can be found in Supplementary S9 and S10.

### 4.3 Sample Preparation and Datasets Acquisition

To construct a training dataset comprising coexisting zebrafish and nanoplastics for HSPEC‐LSFM imaging, we selected transgenic zebrafish (Tg (kdrl: EGFP), sourced from the National Zebrafish Resource Center), and YFP‐labeled nanoplastics (NPs, 0.2 µm, YFP (505/515), Thermo‐Fisher, FluoSpheres carboxylate‐modified microspheres) for co‐embedding. The zebrafish were obtained at 5–7 days post‐fertilization (d.p.f.), euthanized using a 2 g/L tricaine solution, and subsequently fixed in 4% paraformaldehyde. After fixation, the zebrafish were thoroughly rinsed with phosphate‐buffered saline (PBS) and embedded in 1.5% w/w agarose containing the microplastics. The embedded samples were then subjected to UbasM solution [50] for tissue clearing and stored at 4°C in preparation for hyperspectral imaging. All animal procedures were conducted in compliance with the guidelines of the Chinese Council on Laboratory Animal Care.

For the training dataset, low signal‐to‐noise ratio (SNR) hyperspectral light sheet slices were acquired under low illumination power (35 µW) and a short exposure time (30 ms), denoted as “Noisy” images. In contrast, high‐SNR hyperspectral light sheet slices were obtained under high illumination power (58 µW) and long exposure time (300 ms), referred to as high‐SNR images. These fixed/cleared low‐/high‐SNR paired acquisition settings were used for supervised training/reference construction and are summarized together with the live validation and application settings in Supplementary S8.

Here, High‐SNR hyperspectral light‐sheet slices were acquired from the same optical slice under a higher excitation dose and used as paired high‐SNR reference images. These high‐SNR images were treated as experimentally acquired reference approximations rather true noise‐free references. Low‐SNR and high‐SNR images were acquired sequentially using galvanometer scanning while the sample remained stationary, without mechanical repositioning between paired acquisitions; the paired acquisition was typically completed within 10 s. All pairs were registered and screened at the patch level. Patches with residual displacement larger than one pixel or inconsistent landmark correspondence were excluded from the dataset. Pairing fidelity and residual shift distributions are shown in Supplementary S7.

### 4.4 Zebrafish Uptake Nanoplastics Experiment

To investigate the 3D distribution of NPs within the zebrafish, the 5 d.p.f. zebrafish were exposed to the YFP‐labeled NPs solution at a concentration of 5 mg/L (typically from ∼µg/L to ∼g/L in recent research [51, 52, 53]) for 24 h and then returned into DI water. Zebrafish were selected for further imaging at a series of timepoint, which were 12 h (after 12 h exposure, Figure 6a), 24 h (after 24 h exposure, Figures 3a, 4a and 5a), 48 h (after 24 h exposure and 24 h DI water, Figure 6) and 72 h (after 24 h exposure and 48 h DI water, Figure 6). The selected zebrafish were anesthesia with 50 mg/L tricaine solution for 20 min [54] and then embedded in 1.5% w/w agar cylinder for in vivo HSPEC‐LSFM imaging.

For in vivo 3D HSPEC‐LSFM imaging, live zebrafish were acquired under photon‐limited illumination conditions using an excitation power of 58 µW and an exposure time of 30 ms per line. The acquired whole‐fish hyperspectral volumes were cropped into image patches/blocks and processed using the same preprocessing pipeline as that used for paired dataset construction before being enhanced by DsRAN‐NMF. Under the optimized acquisition workflow, whole‐fish 3D hyperspectral sampling could be completed within approximately 28 min. The detailed acquisition settings for all training, validation, and in vivo imaging datasets are summarized in Supplementary S13.

To capture dynamic nanoplastics accumulation within the zebrafish blood circulatory system, 12 d.p.f. specimens exposed to 5 mg/L NPs for 48 h were anesthetized and embedded in 1.5% (w/w) agarose prior to in vivo hyperspectral line‐scanning time‐series acquisition, with 30 µW illumination and 4 ms exposure time per line (0.5 s per frame).

### 4.5 Data Processing

The training dataset underwent rigorous pixel‐level registration and alignment to mitigate displacement caused by environmental vibrations during the data acquisition process. All images were rescaled to standardize the dynamic range by maximum and minimum scaling. Subsequently, the images were cropped into patches of size 256 × 256 × 96 (x, y, λ) for training purposes. The full training dataset consists of 220 pairs of Noisy‐ high‐SNR images. For the reduced‐data training experiment, 55 pairs, 26 pairs, and 13 pairs were selected from the full dataset, representing 25%, 12%, and 6% of the total, respectively. These reduced training subsets were constructed as representative low‐data subsets covering major spatial structures, fluorescence components, signal levels, and noise conditions in the training dataset; they were not intended as repeated random resampling of all possible low‐data partitions. Additionally, 22 pairs of Noisy‐high‐SNR images were set as the test set for network evaluation, ensuring that these images were not involved in the training process.

Train/test partitions were generated at the animal level, and patches from the same image stack were kept within the same split to avoid data leakage. Patches were used for network training and local metric calculation, but were not treated as independent biological replicates for statistical testing. When pixel‐level metrics were calculated, values from patches belonging to the same independent slice/stack were first aggregated before group‐level comparison. Statistical analyses were performed using independent slices/stacks or biological samples as the statistical units, as indicated in Supplementary S8.

In vivo application data underwent identical preprocessing, involving dynamic rescaling and cropping to 256 × 256 × 96, before being fed into the network. The enhanced image patches generated by the network were then stitched to produce the final enhanced image. All image processing operations were completed in MATLAB 2022b.

### 4.6 Training

The network training and predicting process was set in the Python 3.10 environment with framework of PyTorch 2.0 on a Dell T7920 workstation with two Graphics Processing Units (GPUs, NVIDIA A6000, 48 GB), two 2.60 GHz Intel Xeon Gold 6240 CPUs of and 512 GB RAM. For all networks (including DsRAN‐NMF and competitive networks), the training epochs on different datasets were set to 300, and the Adam optimizer was used for optimization. All competing networks' code was directly obtained from their open‐source code.

A multi‐loss‐joint strategy was adopted for DsRAN‐NMF due to its unique architecture to balance the spatial and spectral restoration. The total loss was set as the sum of pixel‐level loss, spatial loss, and spectral loss (i.e., *L_1_*, *L_BCE,_* and *L_SAM_*):

$$L_{\mathrm{total}}=L_{1}+\alpha L_{BCE}+\beta L_{SAM}$$

$$L_{1}=\frac{1}{N}\sum_{i=1}^{N}\left|y_{i}-\hat{y_{i}}\right|$$

$$L_{\mathrm{BCE}}=\frac{1}{N}\sum_{i=1}^{N}\left(y_{i}\times \ln\hat{y_{i}}+\left(1-y_{i}\right)\times \ln\left(1-\hat{y_{i}}\right)\right)$$

$$\;L_{\mathrm{SAM}}=\frac{1}{N}\;\sum_{i=1}^{N}\left(\mathrm{co}s^{-1}\left(\frac{A_{i}\cdot B_{i}}{∥A_{i}∥∥B_{i}∥}\right)\right)$$

where *N* is the number of pixels. *y_i_* the intensity of the high‐SNR image pixel, $\hat{y_{i}}$ the intensity of predicted value. *A_i_* and *B_i_* are the spectra of each pixel in the high‐SNR and predicted images.

### 4.7 Evaluation

For comprehensive performance assessment, this study employed both visualization of denoised hyperspectral data and four established quantitative metrics: Peak Signal‐to‐Noise Ratio (PSNR), Structural Similarity Index (SSIM), Spectral Angle Mapper (SAM), and Spectral Information Divergence (SID). The max intensity projection (MIP) rendering method was typically used for hyperspectral data visualization with the color representing the peak wavelength of the corresponding pixel. The shadow projection employed in some figures was generated by using Imaris 9.0.1. To facilitate visual comparison, image display parameters were individually optimized according to their inherent contrast characteristics through perceptual uniformity calibration.

## Author Contributions

Conceptualization: LC, MZ, RL, KL; methodology: LC, RL, SK, JM, GL, KL; supervision: MZ, LC; visualization: LC, RL; data‐collecting: RL, SW, ZA, YB; Writing – original draft: LC, RL; Writing – review & editing: LC, MZ, SK, JM, SF, TH.

## Funding

This work was supported by the National Key R&D Program of China (2024YFD1700601), National Natural Science Foundation of China (52270008, 52370003).

## Conflicts of Interest

The authors declare no conflicts of interest.

## Supporting information




**Supporting File 1**: advs76802‐sup‐0001‐SuppMat.docx.

**Supporting File 2**: advs76802‐sup‐0002‐MovieS1.mp4.

## Data Availability

All the data that support the findings of this study are available from the corresponding author upon reasonable request. The code is available ahttps://doi.org/10.5281/zenodo.15715786.

## References

[1] J. W. Lichtman and J. A. Conchello , “Fluorescence Microscopy,” Nature Methods 2, no. 12 (2005): 910–919, 10.1038/NMETH817. 16299476

[2] M. J. Wang , Y. Da , and Y. Tian , “Fluorescent Proteins and Genetically Encoded Biosensors,” Chemical Society Reviews 52, no. 4 (2023): 1189–1214, 10.1039/d2cs00419d. 36722390

[3] Z. H. Lei and F. Zhang , “Molecular Engineering of NIR‐II Fluorophores for Improved Biomedical Detection,” Angewandte Chemie International Edition 60, no. 30 (2021): 16294–16308, 10.1002/anie.202007040. 32780466

[4] W. T. Dou , H. H. Han , A. C. Sedgwick , et al., “Fluorescent Probes for the Detection of Disease‐associated Biomarkers,” Science Bulletin 67, no. 8 (2022): 853–878, 10.1016/j.scib.2022.01.014. 36546238

[5] A. A. Gowen , Y. Feng , E. Gaston , and V. Valdramidis , “Recent Applications of Hyperspectral Imaging in Microbiology,” Talanta 137 (2015): 43–54, 10.1016/j.talanta.2015.01.012. 25770605

[6] W. Shi , D. E. S. Koo , M. Kitano , et al., “Pre‐Processing Visualization of Hyperspectral Fluorescent Data with Spectrally Encoded Enhanced Representations,” Nature Communications 11, no. 1 (2020): 726, 10.1038/s41467-020-14486-8. PMC7002680 32024828

[7] D.‐W. Sun , H. Pu , and J. Yu , “Applications of Hyperspectral Imaging Technology in the Food Industry,” Nature Reviews Electrical Engineering 1, no. 4 (2024): 251–263, 10.1038/s44287-024-00033-w.

[8] S. Karim , A. Qadir , U. Farooq , M. Shakir , and A. A. Laghari , “Hyperspectral Imaging: a Review and Trends towards Medical Imaging,” Current Medical Imaging Formerly Current Medical Imaging Reviews 19, no. 5 (2023): 417–427, 10.2174/1573405618666220519144358. 35598236

[9] eds. F.‐J. Kao , G. Keiser , and A. Gogoi , Advanced Optical Methods for Brain Imaging (Springer, 2019), 10.1007/978-981-10-9020-2.

[10] J. Wu , N. Ji , and K. K. Tsia , “Speed Scaling in Multiphoton Fluorescence Microscopy,” Nature Photonics 15, no. 11 (2021): 800–812, 10.1038/s41566-021-00881-0.

[11] Y. Wang , J. Peng , Q. Zhao , Y. Leung , X.‐L. Zhao , and D. Meng , “Hyperspectral Image Restoration via Total Variation Regularized Low‐Rank Tensor Decomposition,” IEEE Journal of Selected Topics in Applied Earth Observations and Remote Sensing 11, no. 4 (2018): 1227–1243, 10.1109/jstars.2017.2779539.

[12] Z. Yong‐Qiang and Y. Jingxiang , “Hyperspectral Image Denoising via Sparse Representation and Low‐Rank Constraint,” IEEE Transactions on Geoscience and Remote Sensing 53, no. 1 (2015): 296–308, 10.1109/tgrs.2014.2321557.

[13] T. Kubo , K. Temma , N. I. Smith , et al., “Hyperspectral Two‐photon Excitation Microscopy Using Visible Wavelength,” Optics Letters 46, no. 1 (2021): 37, 10.1364/OL.413526. 33362007

[14] W. Jahr , B. Schmid , C. Schmied , F. O. Fahrbach , and J. Huisken , “Hyperspectral Light Sheet Microscopy,” Nature Communications 6, no. 1 (2015): 7990, 10.1038/ncomms8990. PMC4569691 26329685

[15] A. J. Bares , M. A. Mejooli , M. A. Pender , et al., “Hyperspectral Multiphoton Microscopy for in vivo Visualization of Multiple, Spectrally Overlapped Fluorescent Labels,” Optica 7, no. 11 (2020): 1587, 10.1364/optica.389982. 33928182 PMC8081374

[16] J. Lever , M. Krzywinski , and N. Altman , “Principal Component Analysis,” Nature Methods 14, no. 7 (2017): 641–642, 10.1038/nmeth.4346.

[17] M. Maggioni , V. Katkovnik , K. Egiazarian , and A. Foi , “Nonlocal Transform‐Domain Filter for Volumetric Data Denoising and Reconstruction,” IEEE Transactions on Image Processing 22, no. 1 (2013): 119–133, 10.1109/tip.2012.2210725. 22868570

[18] A. Buades , B. Coll , and J. M. Morel , “A Non‐Local Algorithm for Image Denoising,” 2005 IEEE Computer Society Conference on Computer Vision and Pattern Recognition (CVPR'05) (IEEE, 2005), 2, 60–65, 10.1109/CVPR.2005.38.

[19] Y. Chang , L. X. Yan , H. Z. Fang , S. Zhong , and W. S. Liao , “HSI‐DeNet: Hyperspectral Image Restoration via Convolutional Neural Network,” IEEE Transactions on Geoscience and Remote Sensing 57, no. 2 (2019): 667–682, 10.1109/TGRS.2018.2859203.

[20] K. X. Wei , Y. Fu , and H. Huang , “3‐D Quasi‐Recurrent Neural Network for Hyperspectral Image Denoising,” IEEE Transactions on Neural Networks and Learning Systems 32, no. 1 (2021): 363–375, 10.1109/TNNLS.2020.2978756. 32217487

[21] D. Yu , Q. Li , X. Wang , Z. Zhang , Y. Qian , and C. Xu , “DSTrans: Dual‐Stream Transformer for Hyperspectral Image Restoration,” 2023 IEEE/CVF Winter Conference on Applications of Computer Vision (WACV), (2023): 3728–3738, 10.1109/WACV56688.2023.00373.

[22] K. X. Li , R. J. Li , G. Y. Li , et al., “SSCDN: a Spatial‐spectral Collaborative Network for Hyperspectral Image Denoising,” Optics Express 32, no. 19 (2024): 32612, 10.1364/OE.532838. 39572984

[23] M. Mickoleit , B. Schmid , M. Weber , et al., “High‐resolution Reconstruction of the Beating Zebrafish Heart,” Nature Methods 11, no. 9 (2014): 919–922, 10.1038/nmeth.3037. 25042787

[24] R. M. Power and J. Huisken , “A Guide to Light‐sheet Fluorescence Microscopy for Multiscale Imaging,” Nature Methods 14, no. 4 (2017): 360–373, 10.1038/nmeth.4224. 28362435

[25] Y. C. Su , H. R. Zhu , K. C. Wong , Y. Chang , and X. T. Li , “Hyperspectral Image Denoising via Weighted Multidirectional Low‐Rank Tensor Recovery,” IEEE Transactions on Cybernetics 53, no. 5 (2023): 2753–2766, 10.1109/TCYB.2022.3208095. 36251897

[26] Y. Chen , J. S. Zeng , W. He , X. L. Zhao , T. X. Jiang , and Q. Huang , “Fast Large‐Scale Hyperspectral Image Denoising via Noniterative Low‐Rank Subspace Representation,” IEEE Transactions on Geoscience and Remote Sensing 62 (2024): 1–14, 10.1109/TGRS.2024.3458395.

[27] J. Y. Cai , W. He , and H. Y. Zhang , “Anisotropic Spatial–Spectral Total Variation Regularized Double Low‐Rank Approximation for HSI Denoising and Destriping,” IEEE Transactions on Geoscience and Remote Sensing 60 (2022): 1–19, 10.1109/TGRS.2022.3202714. PMC12726971 41446555

[28] K. He , X. Zhang , S. Ren , and J. Sun , “Deep Residual Learning for Image Recognition,” 2016 IEEE Conference on Computer Vision and Pattern Recognition (CVPR) (IEEE, 2016): 770–778, 10.1109/CVPR.2016.90.

[29] O. Oktay , J. Schlemper , L. Le Folgoc , et al., “Attention U‐Net: Learning Where to Look for the Pancreas,” Medical Imaging with Deep Learning (MIDL, 2018), 10.48550/arXiv.1804.03999.

[30] Y. X. Wang and Y. J. Zhang , “Nonnegative Matrix Factorization: a Comprehensive Review,” IEEE Transactions on Knowledge and Data Engineering 25, no. 6 (2013): 1336–1353, 10.1109/TKDE.2012.51.

[31] H. Zhao , O. Gallo , I. Frosio , and J. Kautz , “Loss Functions for Image Restoration with Neural Networks,” IEEE Transactions on Computational Imaging 3, no. 1 (2017): 47–57, 10.1109/tci.2016.2644865.

[32] R. Olaf , F. Philipp , and B. Thomas , Medical Image Computing and Computer‐Assisted Intervention – MICCAI 2015, ed. N. Navab , J. Hornegger , W. M. Wells , and A. F. Frangi , (Springer International Publishing, 2015), 234–241.

[33] F. A. Kruse , A. B. Lefkoff , J. W. Boardman , et al., “The Spectral Image Processing System (SIPS)—Interactive Visualization and Analysis of Imaging Spectrometer Data,” Remote Sensing of Environment 44, no. 2‐3 (1993): 145–163, 10.1016/0034-4257(93)90013-N.

[34] C.‐I. Chang , “An Information‐theoretic Approach to Spectral Variability, Similarity, and Discrimination for Hyperspectral Image Analysis,” IEEE Transactions on Information Theory 46, no. 5 (2000): 1927–1932, 10.1109/18.857802.

[35] W. Zhou , A. C. Bovik , H. R. Sheikh , and E. P. Simoncelli , “Image Quality Assessment: from Error Visibility to Structural Similarity,” IEEE Transactions on Image Processing 13, no. 4 (2004): 600–612, 10.1109/TIP.2003.819861. 15376593

[36] H. J. Chiang , D. E. S. Koo , M. Kitano , et al., “HyU: Hybrid Unmixing for Longitudinal in vivo Imaging of Low Signal‐to‐noise Fluorescence,” Nature Methods 20, no. 2 (2023): 248–258, 10.1038/s41592-022-01751-5. 36658278 PMC9911352

[37] X. Gao , X. Huang , Z. Chen , et al., “Supercontinuum‐tailoring Multicolor Imaging Reveals Spatiotemporal Dynamics of Heterogeneous Tumor Evolution,” Nature Communications 15, no. 1 (2024): 9313, 10.1038/s41467-024-53697-1. PMC11522295 39472437

[38] V. Nava , S. Chandra , J. Aherne , et al., “Plastic Debris in Lakes and Reservoirs,” Nature 619, no. 7969 (2023): 317–322, 10.1038/s41586-023-06168-4. 37438590

[39] L. E. Revell , P. Kuma , E. C. Le Ru , W. R. C. Somerville , and S. Gaw , “Direct Radiative Effects of Airborne Microplastics,” Nature 598, no. 7881 (2021): 462–467, 10.1038/s41586-021-03864-x. 34671134

[40] X. Z. Lim , “Microplastics Are Everywhere — But Are They Harmful?,” Nature 593, no. 7857 (2021): 22–25, 10.1038/d41586-021-01143-3. 33947993

[41] H. Bouwmeester , P. C. H. Hollman , and R. J. B. Peters , “Potential Health Impact of Environmentally Released Micro‐ and Nanoplastics in the Human Food Production Chain: Experiences from Nanotoxicology,” Environmental Science & Technology 49, no. 15 (2015): 8932–8947, 10.1021/acs.est.5b01090. 26130306

[42] H. P. Huang , J. Q. Hou , M. X. Li , F. C. Wei , Y. L. Liao , and B. D. Xi , “Microplastics in the Bloodstream Can Induce Cerebral Thrombosis by Causing Cell Obstruction and Lead to Neurobehavioral Abnormalities,” Science Advances 11, no. 4 (2025): adr8243, 10.1126/sciadv.adr8243. PMC11753373 39841831

[43] W. Wang , X. Mao , R. Zhang , et al., “Nanoplastic Exposure at Environmental Concentrations Disrupts Hepatic Lipid Metabolism through Oxidative Stress Induction and Endoplasmic Reticulum Homeostasis Perturbation,” Environmental Science & Technology 57, no. 38 (2023): 14127–14137, 10.1021/acs.est.3c02769. 37683116

[44] A. Kumar , K. E. McNally , Y. Zhang , et al., “Multispectral Live‐cell Imaging with Uncompromised Spatiotemporal Resolution,” Nature Photonics 19, no. 10 (2025): 1146–1156, 10.1038/s41566-025-01745-7. 41048459 PMC12488498

[45] K. Yan , Z. Hu , P. Yu , et al., “Ultra‐photostable Small‐molecule Dyes Facilitate near‐infrared Biophotonics,” Nature Communications 15, no. 1 (2024): 2593, 10.1038/s41467-024-46853-0. PMC10960032 38519530

[46] T. Liu , J. Liu , D. Li , and S. Tan , “Bayesian Deep‐learning Structured Illumination Microscopy Enables Reliable Super‐resolution Imaging with Uncertainty Quantification,” Nature Communications 16, no. 1 (2025): 5027, 10.1038/s41467-025-60093-w. PMC12125172 40447610

[47] R. Shang , M. A. O'Brien , F. Wang , G. Situ , and G. P. Luke , “Approximating the Uncertainty of Deep Learning Reconstruction Predictions in Single‐pixel Imaging,” Communications Engineering 2, no. 1 (2023): 53, 10.1038/s44172-023-00103-1. 38463559 PMC10923550

[48] L. Huang , Y. Li , N. Pillar , T. Keidar Haran , W. D. Wallace , and A. Ozcan , “A Robust and Scalable Framework for Hallucination Detection in Virtual Tissue Staining and Digital Pathology,” Nature Biomedical Engineering 9, no. 12 (2025): 2196–2214, 10.1038/s41551-025-01421-9. PMC12705451 40523934

[49] Y. Li , Y. Su , M. Guo , et al., “Incorporating the Image Formation Process into Deep Learning Improves Network Performance,” Nature Methods 19, no. 11 (2022): 1427–1437, 10.1038/s41592-022-01652-7. 36316563 PMC9636023

[50] L. L. Chen , G. Y. Li , Y. M. Li , et al., “UbasM: an Effective Balanced Optical Clearing Method for Intact Biomedical Imaging,” Scientific Reports 7 (2017): 12218, 10.1038/s41598-017-12484-3. 28939860 PMC5610269

[51] L. Lei , S. Wu , S. Lu , et al., “Microplastic Particles Cause Intestinal Damage and Other Adverse Effects in Zebrafish Danio Rerio and Nematode Caenorhabditis elegans,” Science of the Total Environment 619–620 (2018): 1–8, 10.1016/j.scitotenv.2017.11.103. 29136530

[52] Y. Wāng and Y. Jiang , “Drosophila Melanogaster as a Tractable Eco‐environmental Model to Unravel the Toxicity of Micro‐and Nanoplastics,” Environment International 192 (2024): 109012, 10.1016/j.envint.2024.109012. 39332284

[53] Y. Lu , Y. Zhang , Y. Deng , et al., “Uptake and Accumulation of Polystyrene Microplastics in Zebrafish ( Danio rerio ) and Toxic Effects in Liver,” Environmental Science & Technology 50, no. 7 (2016): 4054–4060, 10.1021/acs.est.6b00183. 26950772

[54] C. Collymore , E. K. Banks , and P. V. Turner , “Lidocaine Hydrochloride Compared with MS222 for the Euthanasia of Zebrafish (Danio rerio),” Journal of the American Association for Laboratory Animal Science 55, no. 6 (2016): 816. 27931323 PMC5113886

