# Supplementary material for: Deep‐Learning‐Driven High‐Fidelity In Vivo Hyperspectral Fluorescence Imaging Under Extreme Photon‐Limited Conditions
Source: Adv Sci (Weinh). 2026 Jul 27;13(59):e76802. doi: 10.1002/advs.76802 (PMC13403730; doi:10.1002/advs.76802)
Supplement: Supplementary file 1 [file ADVS-13-e76802-s002.docx]

**Supplementary Information for**

**Deep-learning-driven high-fidelity in vivo hyperspectral fluorescence imaging under extreme photon-limited condition**

*Renjian Li,^1^ Shutao Wu,^1^ Kaixiang Li^1^ Zhenyu An,^1^ Yuwen Ben,^1^ Guiye LI,^1^ Sunil Kumar, ^2^ James Mcginty,^2^ Tawfique Hasan,^3^ Songnian Fu,^4^ Meng Zhang,^5, *^ LingLing Chen^1, *^*

*^1^College of Health Science and Environmental Engineering, Shenzhen Technology University, Shenzhen, 518118, China.*

*^2^Photonics Group, Department of Physics, Imperial College London, London, SW7 2AZ, United Kingdom.*

*^3^Advanced Electrical Engineering Division, Engineering Department, Cambridge University, CB3 0FA, United Kingdom.*

*^4^ Institute of Advanced Photonics Technology, School of Information Engineering, Guangdong University of Technology, Guangzhou, 51006, China.*

*^5^Advanced Interdisciplinary Institute of Satellite Applications, Beijing Normal University, Beijing, 100875, China.*

**This Word file includes:**

S1. The noise-caused degradation and deep-learning-based recovery of spectral feature space

S2. Optical characterization of confocal line-scanning LSFM for scattering-background rejection

S3. Hyperspectral acquisition preserves spectral separability for zebrafish nanoplastic imaging

S4. Confocal hyperspectral light-sheet fluorescence microscopy system

S5. Disentangling optical and computational contributions

S6. Dual-stream residual attention network combined with non-negative matrix factorization DsRAN-NMF

S7. Pixel-level registration and quality control of paired low-/high-SNR images

S8. Dataset composition, biological sample numbers, and split protocol for all fHSI datasets

S9. Non-negative matrix factorization

S10. Endmember-number selection and robustness analysis of NMF unmixing

S11. Fixed-to-live cross-state validation of DsRAN-NMF

S12. Few-shot transfer learning of DsRAN-NMF

S13. Photon efficiency improvement of DsRAN-NMF for hyperspectral fluorescence imaging

S14. Ablation analysis of DsRAN-NMF model components

S15. Ablation experiment of DsRAN-NMF

S16. Comparison between DsRAN-NMF and DsRAN without NMF in low-data learning

S17. Nanoplastics moving in zebrafish vessels

S18. Performance of DsRAN-NMF in human prostate tissue hyperspectral imaging

S19. Performance of DsRAN-NMF in remote sensing, industrial inspection, and unmanned-aerial-vehicle-based hyperspectral imaging

S20. Zebrafish uptake nanoplastics experiment

S21. Characterization results of the developed imaging platform based on HSPEC-LSFM

S22. In vivo HSPEC-LSFM imaging of zebrafish with sustained exposure to nanoplastics at 48 h and 72 h

S23. Image thresholding segmentation and visualization processing

S24. Quantitative metrics used for model evaluation

S25. Low-data learning HSI denoising results using SOTA deep-learning methods

S26. DsRAN-NMF at varying training data levels

**Other Supplementary Materials**

Movie S1. Flow dynamics of nanoplastics in zebrafish vessels under DsRAN-NMF-enhanced hyperspectral fluorescence microscopy

**S1. The noise-caused degradation and deep-learning-based recovery of spectral feature space.**

Hyperspectral images are invariably corrupted by complex, multi‑source noise introduced during acquisition-such as sensor noise, ambient illumination fluctuations, photon shot noise, and scattering-resulting in nonlinear degradation of the data manifold in spectral feature space. This degradation causes the spectral data distribution to collapse and distort (Fig. S1, “Noisy”), making intrinsic spectrum extraction and endmember identification exceedingly difficult.

Contemporary end‑to‑end deep denoising networks (e.g., HSI‑DeNet, DsTrans, QRNN3D) concentrate on learning local spatial and spectral details but neglect the global geometry of the spectral‑feature space. Although these methods can visually enhance signal‑to‑noise ratio and smooth individual spectral dimensions, they fail to preserve the overall spectral distribution of the scene. As a result, they inherit-or even amplify-the ill-conditioned structure of the noisy spectral space (Fig. S1), causing the restored spectra to lose “unmixability”: they can no longer be reliably associated with real‑world components, undermining the very purpose of hyperspectral imaging.

**Fig. S1. Spectral feature space distribution with PCA dimensionality reduction.** Data points represent individual spectra from the network-testing dataset projected onto the first two principal components. The characteristic V-shaped distribution in the HIGH SNR REFERENCE image corresponds to triple-endmember linear mixing. Under noise corruption, this spectral distribution pattern degrades significantly. While DsRAN-NMF reconstructed HSI successfully restores the HIGH SNR REFERENCE-like distribution in spectral feature space, SOTA methods fail to reproduce the characteristic distribution pattern.

By embedding a nonnegative matrix factorization (NMF) constraint, the DsRAN‑NMF framework is able to efficiently learn and preserve the convex‐geometric distribution of hyperspectral data in spectral feature space (Fig. S1, “HIGH SNR REFERENCE” vs. “DsRAN‑NMF”). This correction not only improves reconstruction accuracy and stability, but also enhances learning efficiency and reduces dependence on extensive training data. Critically, by restoring the global spectral distribution, DsRAN‑NMF yields outputs that are amenable to subsequent spectral unmixing-an outcome of clear practical significance

**S2. Optical characterization of confocal line-scanning LSFM for scattering-background rejection**

To isolate the contribution of the optical front-end, we compared conventional LSFM with the developed confocal line-scanning LSFM using raw zebrafish fluorescence images before any learning-based restoration. Conventional LSFM illuminates an extended light-sheet plane and collects fluorescence from the illuminated region, where residual scattered and in-plane background signals can still contribute to image degradation. In contrast, confocal line-scanning LSFM combines scanned line illumination with slit-confocal detection in a scan-descan geometry, so that fluorescence from the active scan line is preferentially collected while out-of-line background is rejected as illustrated in Fig. 1a.

Fig. S2. Optical principle and raw-data improvement of confocal line-scanning LSFM compared with conventional LSFM. (a) Raw image contrast improvement before deep-learning restoration. (b) Typical contrast comparison between LSFM and confocal line-scanning LSFM.

For comparison, zebrafish brain vasculature and cardiac regions were imaged under matched acquisition conditions using the two imaging modes. Raw images were directly used for contrast analysis. Image contrast was quantified by the normalized root-mean-square contrast:

$$C_{RMS\text{，norm}}=\frac{1}{\overline{I}}\sqrt{\frac{1}{N}\sum_{i=1}^{N} (I_{i}-\overline{I})^{2}}$$

where $I_{i}$is the intensity of the $i$-th pixel, $\bar{I}$is the mean intensity of the analyzed region, and $N$is the total number of pixels

Compared with conventional LSFM, confocal line-scanning LSFM produced visibly clearer vascular and cardiac structures with reduced diffuse background. Quantitatively, the mean C increased from 0.69 in conventional LSFM to 1.59 in confocal line-scanning LSFM, corresponding to an approximately 2.3-fold improvement in raw image contrast. Representative examples showed consistent contrast enhancement in zebrafish brain vasculature and cardiac regions, with individual C values increasing from 0.74 to 1.26, from 0.68 to 1.27, and from 0.76 to 2.79, respectively.

These results demonstrate that the confocal line-scanning LSFM improves the fidelity of the raw fluorescence images by suppressing scattering-induced background and enhancing structural contrast at the acquisition stage. This optical improvement provides higher-quality input data for subsequent hyperspectral detection and DsRAN-NMF restoration, and confirms that the overall fidelity gain of the proposed framework is not solely attributable to post-processing”

**S3. Hyperspectral acquisition preserves spectral separability for zebrafish nanoplastic imaging**

To evaluate whether hyperspectral detection is required for reliable separation of spectrally overlapping fluorescent components, we compared confocal-HSPEC-LSFM with conventional multi-channel LSFM in zebrafish-nanoplastic imaging. This analysis was designed to assess the acquisition-stage contribution of dense spectral sampling before learning-based restoration.

The developed confocal-HSPEC-LSFM integrates scan-descan light-sheet imaging with wavelength SNR reference-resolved detection as illustrated in Fig. 1a. During acquisition, line-scanned fluorescence is de-scanned and coupled into the spectrograph, allowing each scanned spatial position to be recorded with its corresponding emission spectrum. By sequentially scanning across the field of view and through the axial direction, a four-dimensional spatial-spectral data cube is obtained, containing two lateral dimensions, one axial dimension, and one spectral dimension (Fig. S3(a)). This configuration preserves the dense spectral information needed to distinguish EGFP-labelled vasculature, YFP-labelled nanoplastics, and endogenous autofluorescence, which are spatially mixed and spectrally overlapping in live zebrafish imaging.

**Fig. S3. Confocal-HSPEC-LSFM enables raw hyperspectral component separation in zebrafish nanoplastic imaging. (**a) Schematic of the acquired four-dimensional hyperspectral data cube containing spatial and spectral information. (b) Recovered endmember spectra corresponding to EGFP-labelled vasculature, YFP-labelled nanoplastics, and endogenous autofluorescence.

Blind NMF was first applied directly to the raw confocal-HSPEC-LSFM data to examine whether the acquired hyperspectral measurements retained sufficient spectral separability. As shown in Fig 1c, the raw hyperspectral data enabled separation of vascular structures, nanoplastic signals, and autofluorescence background into distinct abundance maps. The corresponding recovered endmember spectra showed characteristic EGFP, YFP, and broad autofluorescence profiles (Fig. S3(b)). This result indicates that confocal-HSPEC-LSFM provides an information-rich spectral representation that can support downstream component separation even before deep-learning restoration.

**Fig. S4. Conventional multi-channel LSFM lacks sufficient spectral sampling for reliable component separation.** (a) Multi-channel LSFM image of zebrafish-nanoplastic signals and representative channel-response profiles showing mixed EGFP, YFP, autofluorescence, and overlap regions. (b) PCA comparison showing a structured spectral distribution in hyperspectral data but poor separability in multi-channel data. (c) Blind NMF applied to multi-channel LSFM data failed to recover biologically meaningful abundance maps and endmember spectra. (d) Channel-number analysis showing that endmember recovery improves with increasing spectral-band number, indicating that dense spectral sampling is required for reliable unmixing.

We next compared this result with conventional multi-channel LSFM using zebrafish-nanoplastic samples with the same fluorescent components. In the multi-channel images, EGFP, YFP-labelled nanoplastics, and autofluorescence produced strongly mixed channel responses (Fig. S4(a)). Representative spectra extracted from different regions showed overlapping and ambiguous intensity profiles, making component assignment difficult. PCA analysis further showed that hyperspectral measurements retained a structured low-dimensional spectral distribution, whereas multi-channel data showed a scattered and poorly separable distribution (Fig. S4(b)). Consistently, blind NMF applied to the multi-channel data failed to recover biologically meaningful abundance maps or endmember spectra (Fig. S4(c)), demonstrating that sparse multi-channel sampling is insufficient for reliable separation of the overlapping fluorescent contributors in this application.

To further examine the role of spectral sampling density, we performed a channel-number analysis by progressively reducing the number of spectral bands used for unmixing. The maximum SAM of the recovered endmembers decreased as the number of retained spectral bands increased, and reliable endmember recovery was achieved only when sufficiently dense spectral sampling was preserved (Fig. S4(d)). These results indicate that the downstream unmixing performance depends not only on computational analysis, but also on whether the acquisition process retains enough spectral degrees of freedom.

Together, these analyses demonstrate that hyperspectral acquisition is essential for preserving the spectral manifold of mixed fluorescent signals in zebrafish-nanoplastic imaging. Conventional multi-channel LSFM lacks sufficient spectral sampling to distinguish EGFP, YFP-labelled nanoplastics, and autofluorescence under strong spectral overlap, whereas confocal-HSPEC-LSFM provides dense spectral information that supports reliable component separation. This acquisition-stage spectral preservation forms the basis for subsequent DsRAN-NMF restoration and downstream hyperspectral unmixing.

**S4. Confocal Hyperspectral light sheet fluorescence microscopy (HSPEC-LSFM).**

The imaging system employed in this study is a self-developed hyperspectral light sheet fluorescence microscopy, which primarily consists of an excitation optical path module and a detection optical path module (Fig. S5). Key components in the excitation path include a 488 nm laser (Cobolt, Skyra), a 2D scanning galvanometer (Thorlabs, GVS212), an F-Theta lens ((Thorlabs, GAS0121), a tube lens (Thorlabs, TTL180-A), and an illumination objective (Olympus, LMPlanFLN, 10x/0.25). The beam emitted from the laser first passes through a bandpass filter (500 nm long pass, Chroma), is shaped and collimated via an aperture, and then enters the galvanometer system. The galvanometer performs high-speed scanning in the X-Y plane to generate a virtual light sheet. This output light sheet sequentially passes through the F-Theta lens, tube lens, and illumination objective arranged in pairwise 4F configurations.

In the excitation optical path, the laser activation/deactivation and power control are managed by custom LabVIEW software following the manufacturer's communication protocol. The laser beam is collimated into a suitable size by expanding system before entering the excitation path's 2D scanning galvanometer. The galvanometer mirrors deflect according to analog voltage signals from an NI-DAQ device to perform scanning. The reflected beam enters the F-Theta lens, which corrects the focal points of beams at different angles onto the same plane. The beam then enters the tube lens for telecentric correction, producing collimated output, and finally passes through the illumination objective, which compresses it into a light sheet with a target thickness of ~5.8 μm.

**Fig.S5. Schematic illustration of the hyperspectral light sheet fluorescence microscopy (HSPEC-LSFM).**

A 4F system formed by the detection objective and tube lens collects the fluorescence signal excited in the sample by the illumination light. This signal then passes through a relay lens group (also in a 4F configuration) and is imaged onto the entrance slit of the hyperspectral spectrometer. To improve photon utilization efficiency, a de-scanning galvanometer is positioned near the Fourier plane of the relay lens group. Synchronized with the excitation galvanometer, it de-scans fluorescence signals from different positions back onto the spectrometer slit, enabling effective light-sheet hyperspectral imaging. This placement of the de-scanning galvanometer in the Fourier plane, synchronized with the excitation galvanometer, achieves line-to-line coupling between the excitation and emission planes (the light-sheet plane). It ensures each excitation beam is synchronously de-scanned into the spectrometer slit while synchronously triggering the spectrometer's camera for 2D plane imaging of the sample. Furthermore, for 3D imaging of the sample, a motorized translation stage moves the sample through different section planes for excitation.

**S5. Disentangling optical and computational contributions**

To clarify the source of performance improvement in the proposed optical-computational framework, we summarized the corresponding control and ablation analyses in Table S1. The framework was divided into two acquisition-level contributions and two restoration-level contributions: scattering rejection by the confocal line-scanning optical front-end, spectral-space preservation by hyperspectral acquisition, spatial/spatial-spectral restoration by the dual-stream DsRAN backbone, and spectral-fidelity preservation by the NMF-guided SAM constraint.

At the optical front-end, conventional LSFM and confocal line-scanning LSFM were compared to evaluate scattering-background rejection. The normalized RMS contrast increased from 0.69 to 1.59, corresponding to an approximately 2.3-fold improvement in raw image contrast. This confirms that confocal line-scanning improves the quality of the acquired data before deep-learning restoration. In parallel, multi-channel LSFM and hyperspectral LSFM were compared to evaluate spectral-space preservation. Multi-channel data showed mixed channel responses, poorly separable PCA distributions, and failed NMF component separation, whereas hyperspectral acquisition preserved structured spectral distributions and supported reliable unmixing.

**Table S1. Summary of ablation analyses disentangling optical and computational contributions.**

| **Tested contribution** | **Optical front-end** | | | **Backbone architecture**  **(DsRAN)** | **NMF-guided SAM constraint** |
| --- | --- | --- | --- | --- | --- |
|  | **Scattering rejection** | **Spectral-space preservation** | |  |  |
| **Ablation / control** | Conventional LSFM vs confocal line-scanning LSFM | | Multi-channel LSFM vs hyperspectral LSFM | Attention U-Net alone / ResNet-101 alone / dual-stream DsRAN | DsRAN without NMF-guided SAM loss vs DsRAN-NMF |
| **Key metric** | Image contrast (C_RMS_) | | PCA distribution; NMF unmixing | SSIM; SAM | SSIM; SAM |
| **Main observation** | Mean contrast increased from 0.69 to 1.59, ~2.3-fold improvement | | Multi-channel data showed mixed responses and failed NMF separation; hyperspectral data preserved structured spectral distributions | Attention U-Net was unstable; ResNet-101 was spatially stable but spectrally limited; dual-stream improved robustness | Dual-stream fusion provides complementary spatial and spatial-spectral restoration |
| **Conclusion** | Confocal LSFM improves raw spatial contrast by suppressing scattering background | | Confocal-HSPEC-LSFM reduces background-induced crosstalk and supports component separation | Dual-stream fusion provides complementary spatial and spatial-spectral restoration | NMF-guided SAM loss improves data efficiency and preserves spectral fidelity |
| **Contribution** | Providing scattering-suppressed, high-quality hyperspectral labels | | | Offering basic restoration ability | Preserving spectral fidelity and hyperspectral manifold structure |

At the computational stage, model ablation was used to evaluate the contribution of the dual-stream backbone and the NMF-guided SAM constraint. Attention U-Net alone was unstable under reduced-data training, while ResNet-101 provided a relatively stable spatial prior but lacked full hyperspectral restoration capability. The dual-stream DsRAN backbone improved restoration robustness by combining spatial and spatial-spectral feature extraction. Further adding the NMF-guided SAM constraint improved data efficiency and preserved spectral fidelity, supporting downstream hyperspectral unmixing.

Together, these analyses show that the observed performance gain does not arise from a single component or from post-processing alone. Instead, confocal line-scanning improves raw spatial contrast, hyperspectral acquisition preserves spectral separability, the dual-stream backbone provides robust restoration, and the NMF-guided SAM constraint maintains the low-dimensional spectral structure required for reliable downstream analysis.

**S6. Dual-****stream residual attention network combined non-negative matrix factorization (DsRAN-NMF).**

The proposed dual-branch architecture processes hyperspectral images (HSI) for enhanced spatial-spectral reconstruction as shown in Fig. S6. Branch 1 (spatial feature extraction branch) employs 2D-convolutional ResNet-101 for deep spatial feature extraction and noise suppression, employing residual blocks to preserve details. Its output features (4×/16× downsampled) are fused and upsampled via a Feature Fusion Module (FFM), generating both rectification features for Branch 2 and full-resolution spatial output for loss calculation. Branch 2 (spatial-spectral feature extraction branch) utilizes a 3D-convolutional Attention U-Net: its encoder (initial 1×1 conv expands channels to 32) progressively reduces spatial resolution while doubling channels, while the decoder employs transposed convolutions and gated attention mechanisms to weight encoder skip-connections. Crucially, spatial skip connections are rectified using Branch 1 features to enhance critical details. The final 1×1 conv outputs reconstructed HSI. This synergistic design captures spatial structure (Branch 1) and spectral-spatial features (Branch 2), optimized through dual loss functions for robust reconstruction.

As shown in Fig. S7, the Feature Fusion Module (FFM) integrates multi-scale abstract features (a1: 16× downsampled, a2: 4× downsampled) from ResNet-101, Both features undergo convolution-batch norm-ReLU (CBR) blocks for enhanced extraction and channel reduction. Feature a1 is then upsampled 4× via bilinear interpolation to match a2's spatial dimensions. The aligned features are concatenated, combining shallow and deep representations. This fused output passes through an RCBL residual module for preliminary feature refinement and noise filtering while maintaining low parameterization. The resulting feature b1 is upsampled 4× (bilinear), processed by a 3 × 3 convolution to generate b2 (full spatial resolution), and finally reduced to 2D spatial features b3 via a 1 × 1 convolution.

**Fig.S6. The architecture of DsRAN-NMF network.** A dual-stream approach is employed in this model: the first path utilizes ResNet-101 with 2D convolutions for deep spatial feature extraction, followed by feature fusion and up-sampling via a Feature Fusion Module (FFM). The second path, based on an Attention U-Net with 3D convolutions, extracts spectral-spatial features and rectifies its spatial skip connections using the feature grid generated by the first path to enhance critical details. The network's reconstructed output and the High SNR reference (HIGH SNR REFERENCE) undergo Non-negative Matrix Factorization (NMF) for dimensionality reduction, enabling spectral loss computation.

**Fig.S7. The architecture of Feature Fusion Module (FFM).** The FFM integrates multi-scale ResNet-101 features (16×/4× downsampled) through CBR-enhanced processing. After aligning dimensions via bilinear upsampling, features are concatenated and refined through a parameter-efficient RCBL module. Final full-resolution 2D features are generated through sequential upsampling and convolution operations.

**S7. Pixel-level registration and quality control of paired low-/high-SNR images**

To construct paired training data for supervised restoration, low-SNR and high-SNR hyperspectral images were acquired sequentially from the same optical slice. Because even small spatial offsets between paired images can bias pixel-wise training and cause blurred reconstruction of small punctate nanoplastic signals, all paired images were registered and screened before being included in the training and evaluation datasets.

For each low-/high-SNR pair, the hyperspectral data were first converted to 2D intensity projections by summing or averaging the spectral channels. Rigid translational registration was then performed using phase-correlation-based shift estimation between the low-SNR and high-SNR intensity images. The estimated x- and y-shifts were applied to the full hyperspectral data cube so that all spectral channels were translated consistently. After this global pixel-level alignment, local vascular structures and punctate nanoplastic signals were visually inspected as landmarks to confirm the spatial correspondence between the paired low-SNR input and high-SNR reference. Pairs with residual displacement larger than 1-pixel, obvious local mismatch, or inconsistent landmark correspondence were excluded from the dataset.

**Fig. S8. Pixel-level registration and residual-shift analysis of paired low-/high-SNR images.** (a) Representative low-SNR and high-SNR reference image patches after registration. Local vascular and punctate nanoplastic landmarks show close spatial correspondence after alignment. Enlarged ROIs show within-pixel residual displacement between paired acquisitions. (b) Distribution of residual x- and y-shifts after registration, estimated by phase-correlation-based shift analysis. Most retained paired patches showed residual displacement within one pixel. (c) Distribution of raw x- and y-shifts before registration, showing broader displacement between paired low-/high-SNR acquisitions before correction.

Representative paired images after registration are shown in Fig. S8(a). The enlarged regions demonstrate that corresponding punctate structures remained aligned within approximately one pixel after correction. To quantify pairing fidelity, the residual x- and y-displacements of retained patches were further estimated using the same phase-correlation-based shift analysis. After registration and screening, most retained paired patches showed residual displacement within one pixel (Fig. S8(b)). In comparison, the raw paired images before registration showed a broader shift distribution, although most original offsets were still within approximately two pixels (Fig. S8(c)).

These results confirm that the paired dataset was constructed with strict registration quality control. The high-SNR images were therefore used as experimentally acquired reference approximations for network training, rather than true noise-free High SNR reference. Although residual photobleaching, spectral drift, or sub-pixel mismatch cannot be fully eliminated in experimental fluorescence imaging, the short sequential acquisition, stationary sample configuration, phase-correlation registration, and patch-level screening minimized pairing errors and provided sufficiently aligned low-/high-SNR references for training DsRAN-NMF.

**S8. Datasets composition, biological sample numbers and split protocol for all fHSI datasets.**

**Table S2. Dataset composition, biological sample numbers, and split protocol for training and evaluation.**

| **Dataset / task** | | **Uses** | **Patches** | **Independent slices** | **Biological samples** |
| --- | --- | --- | --- | --- | --- |
| Train datasets-Fixed/cleared zebrafish-NP paired fHSI | 100% | Training | 220 | 123 | 41 |
|  | 25% | Training | 55 | 52 | 32 |
|  | 12% | Training | 26 | 25 | 13 |
|  | 6% | Training | 13 | 13 | 7 |
| Test datasets-Fixed/cleared zebrafish-NP paired fHSI | | Testing (Fig.4) | 22 | 22 | 8 |
| Train datasets-live zebrafish-NP paired fHSI (6%) | | Training | 13 | 13 | 7 |
| Test datasets-live zebrafish-NP paired fHSI | | Testing (Fig.R6) | 29 | 29 | 6 |
| Slice HSI data | | Evaluating the restoration of fig3. (b) | 5 | 1 | 1 |
| Transfer datasets-2D HSI | | Transfer training | 6 | 2 | 2 |

Animal-independent train/test; patches from same stack kept in same split; 2D slice HSI data was collected independently

**S9. Non-negative matrix factorization.**

In this paper, hyperspectral images are represented as a non-negative matrix V, which is factorized via Non-negative Matrix Factorization (NMF) into a basis matrix W (denoting prototype spectra) and a coefficient matrix H (denoting the abundance of each pixel with respect to these spectra), thereby achieving dimensionality reduction and extraction of interpretable low-dimensional features from high-dimensional spectral data. Here, hyperspectral data is regarded as a non-negative matrix V with the size of n × m, where n is the number of spectral bands and M is the number of pixels. The non-negative matrix W with size n × r and non-negative matrix H with size r × m is found by unmixing algorithm, which satisfies $V\approx W\times H$or:

$$V_{nm}\approx(WH)_{nm}=\sum_{a=1}^{r} W_{na}H_{am}$$

$$\min f\text{(}W,H\text{)}=\frac{1}{2}\text{ }\left\|V-WH\right\|_{F}^{2} (W\geq0\text{,}H\geq0\text{,}1_{r}^{T}H=1_{m}^{T})$$

Since NMF minimizes || V - WH ||2 without relying on any labeled information, it functions both as a prototypical matrix factorization algorithm and as an unsupervised learning method; the non-negativity constraint ensures that the extracted basis spectra possess physical interpretability and sparsity, enabling their direct use in subsequent classification, clustering, or target recognition tasks.

NMF is typically solved via multiplicative update (MU) rules, alternating nonnegative least squares (ANLS), or gradient‐/coordinate‐descent algorithms. Integrating NMF into end‐to‐end neural‐network architectures usually requires that each update step be differentiable so that gradients with respect to a reconstruction‐loss can flow back. Although gradient‐descent-based NMF algorithms (e.g., projected gradient methods) are inherently differentiable, implementing their update steps as internal PyTorch operations can inadvertently detach the computation graph-causing gradients to be released too early and preventing them from flowing back into the main network. Here, the implement of MU updates as a sequence of element‐wise multiplications and divisions, which can be unrolled in a computational graph and therefore supports gradient backpropagation. Approximate factorization W and H are found by iteration of standard multiplicative rule for non-negative V with random nonnegative initial conditions:

$$W\leftarrow W.*(VH^{T})./(WHH^{T}); H\leftarrow H.*(W^{T}V)./(W^{T}WH)$$

$$W\geq0\text{,}H\geq0\text{,}1_{r}^{T}H=1_{m}^{T}$$

NMF computations were implemented within the PyTorch framework, with all matrices represented as torch.Tensor objects to guarantee that gradients are correctly tracked and preserved throughout backpropagation.

**S10. Endmember-number selection and robustness analysis of NMF unmixing**

To justify the choice of endmember number in blind NMF unmixing, we performed a rank-selection and robustness analysis on representative zebrafish-nanoplastic hyperspectral data. The expected fluorescent contributors in this imaging task were EGFP-labelled vasculature, YFP-labelled nanoplastics, and endogenous autofluorescence. However, because weak residual background or spectral variability may introduce additional low-amplitude components, the NMF rank was not determined solely by prior biological knowledge. Instead, we evaluated the appropriate rank using intrinsic-dimensionality estimation, reconstruction residuals, spectral separability metrics, and representative unmixing results across different ranks.

**Fig. S9. Rank-selection analysis for NMF unmixing of zebrafish-nanoplastic hyperspectral data. (a)** Eigenvalue-based rank estimation. Multiple intrinsic-dimensionality criteria, including eigen-gap, scree-elbow, HFC, NWHFC, HySime, and VD, indicated a dominant spectral dimensionality around 3-4 components. **(b)** Spectral stability and separability across NMF ranks. Intra-class SAM was used to evaluate the stability of matched endmembers, while inter-class SAM was used to evaluate separation between different recovered components. **(c)** Relative reconstruction residual as a function of NMF rank. The residual decreased rapidly up to approximately k = 3-4 and then showed a slower reduction trend. **(d)** Representative k = 2 unmixing result showing under-separation, where spectrally distinct components were merged. **(e)** Representative k = 4 unmixing result showing separation of the main EGFP-, YFP-nanoplastic-, and autofluorescence-related components together with an additional residual/background-like component.

First, eigenvalue-based rank estimation was applied to the hyperspectral data. Multiple intrinsic-dimensionality criteria, including eigen-gap, scree-elbow, HFC, NWHFC, HySime, and VD, suggested a dominant spectral dimensionality around 3-4 components (Fig. S9(a)). This range was consistent with the expected EGFP/YFP/autofluorescence contributors, with the possible presence of an additional residual background component. We then swept the NMF rank from k = 2 to k = 8. Reconstruction residuals decreased rapidly when the rank increased from 1 to approximately 3-4, and then approached a slower reduction trend at higher ranks (Fig. S9(c)), indicating that most of the explainable spectral structure was captured by a small number of components. In parallel, intra-class and inter-class SAM were calculated to evaluate spectral stability and separability across ranks (Fig. S9(b)). The intra-class SAM measured the consistency of matched endmembers across repeated runs, whereas the inter-class SAM measured the spectral separation between different recovered endmembers. The rank-sweep analysis showed that k = 3-4 provided a reasonable balance between reconstruction accuracy, endmember stability, and spectral separability.

Representative unmixing results further supported this choice. When k = 2, spectrally distinct contributors were forced to merge into the same components, leading to under-separation of the EGFP/YFP/autofluorescence signals (Fig. S9(d)). In contrast, k = 4 separated the main biologically interpretable components while additionally extracting a residual/background-like component (Fig. S9(e)). Importantly, the primary EGFP-, YFP-nanoplastic-, and autofluorescence-related spectra remained consistent when the rank varied within the reasonable range. Therefore, rank = 3 was used as the default biologically interpretable model for the main analysis, while the rank-sweep results indicate that the main unmixing conclusions were not dependent on a single arbitrary rank choice.

We further evaluated the sensitivity of NMF unmixing to random initialization (Table S3 and Fig.S10). Blind NMF was repeated 10 times using independent random initializations with rank fixed at 3. For each run, the recovered spectra were matched to the corresponding reference endmembers according to the minimum spectral angle mapper (SAM), and the spectra were normalized for comparison. Across all repetitions, the recovered EGFP-, YFP-nanoplastic-, and autofluorescence-related endmembers converged to highly similar spectral profiles (Fig. S10). The mean SAM values were 0.0433, 0.0532, and 0.0368 for the three reference endmembers, respectively, indicating low run-to-run variation. These results demonstrate that the NMF unmixing was stable to random initialization and that the recovered dominant endmembers were reproducible under the tested conditions.

Together, these analyses support the use of NMF-based linear unmixing for separating the dominant fluorescent contributors in this study. The selected rank was guided by biological prior knowledge and validated by eigenvalue-based dimensionality estimation, rank-sweep reconstruction behavior, spectral separability metrics, and random-initialization stability. This does not imply that linear NMF fully captures all possible nonlinear light-tissue interactions, but it shows that the main EGFP/YFP-nanoplastic/autofluorescence separation is stable under the present confocal-HSPEC-LSFM acquisition and analysis conditions.

**Table S3. Stable three-endmember recovery over 10 random NMF initializations.**

|  | Repeat times | | | | | | | | | |  |
| --- | --- | --- | --- | --- | --- | --- | --- | --- | --- | --- | --- |
| epoch | 1 | 2 | 3 | 4 | 5 | 6 | 7 | 8 | 9 | 10 | average |
| Randn initial value  (mean SAM) | 0.06 | 0.05 | 0.05 | 0.05 | 0.05 | 0.05 | 0.05 | 0.05 | 0.05 | 0.05 | 0.052±0.002 |

**Fig. S10. Initialization robustness of NMF endmember recovery.** Blind NMF was performed with rank = 3 and repeated 10 times using independent random initializations. The recovered endmember spectra from each run were matched to the corresponding reference endmembers using the minimum SAM criterion and normalized for visualization. The close overlap of the recovered spectra and the low mean SAM values indicate stable three-endmember recovery across random initializations.

**S11. Fixed-to-live cross-state validation of DsRAN-NMF**

A key concern in applying DsRAN-NMF to live zebrafish imaging is whether a model trained on fixed/cleared paired samples can generalize to live in vivo data. Fixed/cleared samples are advantageous for constructing accurately paired low-/high-SNR training references because they minimize motion-induced mismatch during sequential acquisition. However, live zebrafish differ from fixed/cleared specimens in physiological state, residual motion, local scattering environment, and biological heterogeneity. We therefore performed a direct fixed-to-live cross-state validation to evaluate whether the fixed-trained model retained restoration fidelity when applied to live zebrafish-nanoplastic fHSI data acquired under the same confocal-HSPEC-LSFM configuration.

For this validation, a new live-to-live paired fHSI dataset was acquired from live zebrafish exposed to YFP-labelled nanoplastics. For each field of view, photon-limited live images were acquired using 4 μW and 15 ms, and paired high-SNR live references were acquired from the same optical slice using 632 μW and 100 ms. The paired live data were registered and screened using the same preprocessing workflow as the fixed/cleared paired dataset, including intensity normalization, patch cropping, and pixel-level alignment. The live dataset was divided into a live-training (6%) subset containing 13 paired patches from 13 independent slices and 7 biological samples, and an independent live-test subset containing 29 paired patches from 29 independent slices and 6 biological samples. Patches from the same image stack or animal were kept within the same split to avoid data leakage.

Two models were then compared on the same live-test dataset. The first model was trained only on the fixed/cleared zebrafish-nanoplastic paired fHSI dataset and was directly applied to the live-test data without retraining. The second model was trained using the live-training subset acquired under the same live imaging configuration. Both models used the same DsRAN-NMF architecture, preprocessing pipeline, training schedule, and evaluation metrics. Restoration performance was evaluated by visual comparison, line-profile analysis, and quantitative metrics including PSNR, SSIM, and SAM against the paired high-SNR live references.

As shown in Fig. S11(a), the fixed-trained DsRAN-NMF restored live zebrafish images with clearer vascular structures and reduced background noise compared with the noisy input. The restored structures and intensity profiles were comparable to those obtained using the live-trained model and were close to the paired high-SNR reference. Quantitatively, both the fixed-trained and live-trained models substantially improved restoration quality relative to the noisy live input (Fig. S11x(b)). The noisy data showed low PSNR of approximately **~**12 dB and SSIM ~0.1, whereas the fixed-trained and live-trained models increased PSNR to ~31 dB and SSIM to ~0.75. Spectral fidelity was also improved, with SAM reduced from approximately ~0.78 in the noisy input to ~0.20-0.36 after restoration. These results indicate that the fixed-trained model retains effective restoration performance on live zebrafish fHSI data when the imaging platform, fluorophore composition, and photon-limited degradation conditions are matched.

**Fig. S11. Fixed-to-live cross-state validation and spectral-manifold comparison of zebrafish fHSI data. (a)** Representative live-data restoration results from noisy input, live-trained DsRAN-NMF, fixed-trained DsRAN-NMF, and paired high-SNR reference, with corresponding intensity-profile comparison. **(b)** Quantitative comparison of PSNR, SSIM, and SAM on the live-test dataset. Both fixed-trained and live-trained DsRAN-NMF improved restoration quality relative to the noisy input. **(c)** Representative live-HSI and fixed-HSI images showing comparable zebrafish vasculature and nanoplastic signal characteristics. **(d)** Separate PCA distributions of live-HSI and fixed-HSI spectra. **(e)** Merged PCA projection of live-HSI and fixed-HSI spectra, where spectra from both datasets were jointly projected into the same PCA space to evaluate whether the two sample states occupy overlapping spectral distributions.

We further examined whether the fixed/cleared and live datasets occupied similar spectral domains. Representative live-HSI and fixed-HSI images showed comparable EGFP-labelled vasculature, YFP-labelled nanoplastic signals, and background autofluorescence patterns (Fig. S11(c)). PCA analysis was performed on spectra sampled from the live and fixed/cleared datasets. When analyzed separately, both datasets showed structured low-dimensional spectral distributions (Fig. S11(d)). In the merged PCA analysis, spectra from live-HSI and fixed-HSI datasets were jointly projected into the same PCA space. The merged distribution formed an overlapping bounded spectral manifold rather than two clearly separated preparation-specific clusters (Fig. S11(e)), suggesting that the two datasets preserve similar spectral-variation directions and endmember-mixing geometry.

Together, these results support the use of fixed/cleared paired data for training DsRAN-NMF in this zebrafish-nanoplastic imaging task, while also defining the scope of the generalization claim. The validation does not imply unrestricted transfer across arbitrary biological specimens, fluorophore combinations, or imaging systems. Instead, it demonstrates that DsRAN-NMF can transfer from fixed/cleared paired zebrafish-nanoplastic training data to live zebrafish uptake/circulation imaging under the same confocal-HSPEC-LSFM platform, similar fluorophore composition, and comparable photon-limited fHSI degradation conditions. This result is consistent with the design of the NMF-guided SAM constraint, which encourages restoration within a compact low-dimensional spectral manifold and helps preserve the endmember-mixing geometry required for downstream unmixing.

**S12. Few-shot transfer learning of DsRAN-NMF.**

Two-dimensional (2D) line-scanning hyperspectral fluorescence images and three-dimensional (3D) light-sheet hyperspectral images differ not only in dimensionality but also in imaging characteristics-such as the prevalence of defocus and scattering artifacts in 2D acquisition. As a result, denoising networks trained exclusively on 3D hyperspectral volumes cannot be directly applied to 2D datasets. However, transfer learning offers a promising strategy to leverage the spectral feature extraction and noise discrimination priors learned from 3D datasets, thereby reducing the demand for large-scale labeled training data in the 2D domain.

In this study, we transfer a DsRAN-NMF network-pretrained on 13 paired 3D hyperspectral slices-using only six noisy-clean 2D hyperspectral image pairs. As shown in Fig. S5, the training loss declined rapidly within the first 60 epochs and plateaued after approximately 120 epochs. We selected two representative checkpoints-epoch 60 (early inflection point) and epoch 175 (lowest loss)-to generate predictions. The corresponding visual results (Fig. S12 (a)) and quantitative metrics (Fig. S12 (c)) demonstrate that the network had already achieved near-optimal performance by epoch 60, with SSIM and SAM values approaching those obtained at epoch 175.

These findings suggest that transfer learning enable substantial denoising performance on 2D hyperspectral fluorescence images with minimal training data (6 pairs) and computational cost (~60 epochs, ~10 minutes). This approach significantly enhances the generalizability of deep hyperspectral denoising models and broadens their applicability across diverse imaging modalities.

**Fig.S12. Results of few-shot transfer learning of DsRAN-NMF.** (a) Visualization of 2D line-scanning hyperspectral fluorescence images before/after DsRAN-NMF enhancement with few-shot transfer learning strategy. (b) Loss variation of few-shot transfer learning process. (c) SSIM and SAM of transfer learning DsRAN-NMF at 60 and 175 epochs.

**S13. Photon efficiency improvement of DsRAN-NMF for hyperspectral fluorescence imaging.**

To provide a standardized and directly comparable photon-efficiency calculation, we summarized the acquisition parameters used for different fHSI datasets in Table S4. These datasets were acquired for different purposes, including supervised paired-data construction, in vivo biological application, 2D HSI imaging, and live-to-live photon-efficiency validation. Therefore, acquisition settings from different datasets should not be directly mixed to define a unified photon-efficiency gain.

In this work, photon efficiency was defined as the reduction in excitation dose required to achieve comparable restoration quality under the same imaging task and sample condition. Because the excitation wavelenHigh SNR referenceh, optical path, detector, and sample type were fixed within the live-to-live validation experiment, the delivered excitation dose was approximated as proportional to the product of excitation power and exposure time:

$$\eta_{\mathrm{gain}}=\frac{P_{\mathrm{ref}}\times t_{\mathrm{ref}}}{P_{\mathrm{low}}\times t_{\mathrm{low}}}$$

where $P_{\mathrm{ref}}$and $t_{\mathrm{ref}}$denote the excitation power and exposure time used for live high-SNR reference acquisition, respectively, and $P_{\mathrm{low}}$and $t_{\mathrm{low}}$denote the excitation power and exposure time used for photon-limited live acquisition followed by DsRAN-NMF restoration.

For the standardized live-to-live validation dataset, the live high-SNR reference was acquired using 632 μW excitation power and 100 ms exposure time, whereas the photon-limited live acquisition was acquired using 4 μW excitation power and 15 ms exposure time. Therefore, the photon-efficiency gain was calculated as:

$$\eta_{\mathrm{gain}}=\frac{632\times100}{4\times15}=\frac{63200}{60}\approx1053$$

Thus, the photon-limited acquisition used approximately 1053-fold lower excitation dose than the live high-SNR reference acquisition. This controlled live-to-live comparison provides the experimental basis for the statement of up to three orders-of-magnitude improvement in photon efficiency.

The fixed/cleared paired dataset was used for supervised training/reference construction and was not used as the primary basis for the standardized photon-efficiency calculation. Similarly, the in vivo 3D HSPEC-LSFM and in vivo 2D HSI imaging settings were used for biological application experiments rather than for defining the controlled photon-efficiency gain. By separating these acquisition settings and using the new live-to-live validation dataset for dose comparison, the photon-efficiency estimate avoids mixing static paired-restoration conditions with in vivo application conditions.

**Table S4. Standardized acquisition parameters for all fHSI datasets.**

| **Experiment** | **Sample** | **Purpose** | **Power** | **Exposure** |
| --- | --- | --- | --- | --- |
| Noisy training dataset^1^ | fixed/cleared zebrafish | model training | 35 μW | 30 ms |
| High-SNR reference^1^ | fixed/cleared zebrafish | paired reference | 58 μW | 300 ms |
| *In vivo* 3D HSPEC-LSFM^1^ | live zebrafish | biological application | 58 μW | 30 ms |
| *In vivo* 2D HSI imaging^2^ | live zebrafish | biological application | 0.1 mW | 100 ms |
| New live-to-live low-SNR^3^ | live zebrafish | standardized photon-efficiency validation | 4 μW | 15 ms |
| New live-to-live high-SNR^3^ | live zebrafish | high-SNR reference | 632 μW | 100 ms |
| *In vivo* 3D HSPEC-LSFM^3^ | live zebrafish | Speed testing | 6 μW | 10 ms |

1, Acquired using the confocal-HSPEC-LSFM system equipped with a Princeton Instruments IsoPlane-Fergie module. 2, Acquired using a line-scanning hyperspectral imaging system coupled to a Nikon Ti2-U microscope. 3, Acquired using the optimized confocal-HSPEC-LSFM system equipped with a Princeton Instruments IsoPlane SCT 320 module.

**Table S5. Acquisition-throughput parameters for upgraded whole-fish 3D confocal-HSPEC-LSFM imaging.**

| exposure/frame (ms) | scan number | time per slice （s） | stack number |
| --- | --- | --- | --- |
| 10 | 375 | 3.75 | 3 |
| slice number | time per stack(min) | whole time (min) | |
| 150 | 9.375 | 28.125 | |

**S14. Ablation analysis of DsRAN-NMF model components**

To evaluate the contribution of each computational component in DsRAN-NMF, we performed model ablation using the same paired dataset, train/test split, data-reduction ratios, and training schedule. The tested variants included Attention U-Net alone, Encoder+ResNet-101 as the 2D spatial branch, dual-stream DsRAN without the NMF layer, and the full DsRAN-NMF model. Loss terms were matched to the output of each variant: L1 loss was used for hyperspectral reconstruction, BCE loss was used for the 2D spatial branch, and L1+BCE loss was used for dual-stream DsRAN. Therefore, the comparison between DsRAN without the NMF layer and full DsRAN-NMF isolates the contribution of the NMF-guided SAM loss.

The ablation results show that Attention U-Net alone was unstable under reduced training data, with SSIM decreasing from 0.7309 to 0.3550 at 25% training data and SAM remaining high across all data regimes. Encoder+ResNet-101 provided relatively stable spatial restoration but did not directly support hyperspectral SAM evaluation as a 2D branch. Dual-stream DsRAN improved the overall restoration performance, reaching SSIM = 0.8187 and SAM = 0.2168 with the full dataset, but its spectral fidelity became less stable under reduced-data settings. In contrast, full DsRAN-NMF maintained stable SSIM values around 0.82 and low SAM values around 0.21-0.22 across all training sizes from 100% to 6%. These results indicate that the dual-stream architecture improves restoration robustness, while the NMF-guided SAM constraint further preserves spectral fidelity and enhances data efficiency under limited paired training data. More details can be seen in S15 and S16

**Table. S6. Model variants and matched loss settings for DsRAN-NMF ablation analysis.**

| **Model variant** |  | ***SSIM*** | | | | ***SAM*** | | | |
| --- | --- | --- | --- | --- | --- | --- | --- | --- | --- |
| **Dataset size** | **Loss** | **100%** | **25%** | **12%** | **6%** | **100%** | **25%** | **12%** | **6%** |
| Attention U-Net (backbone) | L1 | 0.7309 | 0.3550 | 0.4162 | 0.4094 | 0.5195 | 0.6073 | 0.5231 | 0.5096 |
| Encoder+ResNet-101 (2D branch) | BCE | 0.7283 | 0.6701 | 0.7104 | 0.6962 | N.A. | N.A. | N.A. | N.A. |
| DsRAN without NMF layer | L1+BCE | 0.8187 | 0.7142 | 0.6854 | 0.7211 | 0.2168 | 0.2322 | 0.5347 | 0.3332 |
| Full DsRAN-NMF | L1+BCE+L_SAM_ | 0.8269 | 0.8232 | 0.8227 | 0.8238 | 0.2082 | 0.2127 | 0.2216 | 0.2239 |
| Loss terms were matched to each model output: L1 for hyperspectral reconstruction, BCE for the 2D spatial branch, and L1+BCE for the dual-stream DsRAN. The comparison between DsRAN without NMF layer (L1+BCE) and full DsRAN-NMF (L1+BCE+L_SAM) isolates the contribution of the NMF-guided SAM loss. | | | | | | | | | |

**S15. Ablation experiment of DsRAN-NMF.**

We performed ablation studies using Attention U-Net and ResNet-101 as standalone backbone architectures trained under identical datasets to validate the efficacy of the DsRAN-NMF framework. Both networks were evaluated with progressively reduced training sets (100%, 25%, 12%, and 6% of 220 pairs) to comprehensively assess their fully supervised characteristics. At 100% training data, Attention U-Net demonstrated unstable image restoration quality (SSIM: 0.73 ± 0.2762, range: 0.07-0.97) and poor spectral recovery (SAM: 0.5196 ± 0.0887). While ResNet-101 showed relatively stable spatial reconstruction, its overall performance remained mediocre. Qualitatively, Attention U-Net outputs exhibited significant artifacts and spectral distortions, whereas ResNet-101 reconstructions suffered from critically low signal-to-noise ratios in 2D restorations. These results confirm that neither architecture alone can effectively handle complex hyperspectral denoising tasks. The dual-stream DsRAN structure, which integrates both backbones through feature fusion, substantially enhances denoising capability.

**Fig. S13. The results of ablation experiment of DsRAN-NMF.** The changes of SSIM and SAM of Attention U-net, DsRAN-NMF and ResNet-101 and 2D slices image before and after restoration using networks (100% datasets). Color bar: 500-520 nm.

During progressive training data reduction, Attention U-Net exhibited sharp deterioration in spatial restoration quality while maintaining consistently poor spectral performance. In contrast, ResNet-101's 2D reconstruction showed minimal degradation, likely attributable to the lower complexity of spatial restoration compared to spectral recovery. This differential response further demonstrates how the DsRAN-NMF architecture enhances network robustness and reduces dependence on large, high-quality training datasets. The feature fusion mechanism effectively compensates for individual backbone limitations: while Attention U-Net becomes increasingly unstable with data scarcity, and ResNet-101 lacks spectral discrimination capacity, their synergistic integration maintains consistent performance across data regimes.

These findings reveal a critical architectural advantage beyond the NMF constraint discussed previously. Where NMF provides physical regularization at the feature level, the dual-stream design creates intrinsic error correction at the structural level. ResNet-101 provides stable spatial priors that ground Attention U-Net's spectral attention mechanisms, preventing hallucinated features when training data is sparse. This hierarchical robustness explains DsRAN-NMF's exceptional performance at minimal (6%) training levels - an achievement unattainable by either standalone backbone. The architecture fundamentally redefines data efficiency in hyperspectral restoration by transforming competing deficiencies into complementary strenHigh SNR referencehs through learned feature fusion.

**S16. Comparison between DsRAN-NMF and DsRAN without NMF in low-data learning.**

To demonstrate the critical role of the NMF constraint under limited training data, we performed an ablation study by removing the NMF module while retaining the DsRAN architecture. Figure S8 shows this modified DsRAN’s performance when trained with only 25%, 12%, and 6% of the original dataset. Reducing training data to 25% caused a sharp deterioration in spatial reconstruction quality (SSIM), while further reduction to 12% led to a drastic decline in spectral fidelity (SAM). This confirms the DsRAN architecture’s inherent dependence on substantial training data.

The NMF constraint effectively mitigates this data dependency, enabling robust training on significantly reduced datasets. Mechanistically, NMF operates as a low-rank representation constraint that rapidly extracts authentic spectral features from complex noise patterns. This reduces the network’s data requirements while enhancing robustness against data scarcity. The non-negativity constraint inherently enforces physical plausibility in hyperspectral reconstructions, preventing unphysical artifacts while promoting noise suppression through sparse representations. Consequently, DsRAN-NMF exhibits superior adaptability to diverse noise types and scenes.

**Fig. S14. The changes of SSIM and SAM metrics of DsRAN-NMF and DsRAN without NMF by gradually decreasing the training data size (25%, 12%, 6%).**

Theoretically, integrating the NMF-derived loss function achieves two advantages: 1) enhanced denoising efficacy through intrinsic dimensionality reduction and spectral unmixing principles, and 2) faithful preservation of physical-spectral characteristics. This synergistic mechanism yields precise and stable denoising outcomes even under extreme data constraints.

**S17. Nanoplastics moving in zebrafish vessels.**

After prolonged circulation, nanoplastic signals were observed in the distal caudal vasculature of zebrafish. As shown in Fig. S15, DsRAN-NMF-enhanced hyperspectral imaging enabled time-lapse visualization of nanoplastic dynamics in the tail vessels, with nanoplastic particles shown in green and vessels shown in blue. In the orange dashed region, the nanoplastic signal remained spatially persistent over consecutive frames, suggesting local vessel-associated retention or aggregation. By contrast, the yellow dashed region showed a transient nanoplastic signal that disappeared within the next time frame, indicating rapid particle passage through the microvasculature. These results illustrate the ability of the proposed imaging framework to separate relatively static nanoplastic accumulation from dynamic circulating particles in vivo.

**Fig. S15. Time-lapse hyperspectral fluorescence imaging of nanoplastic circulation and vessel-associated accumulation in the zebrafish tail after prolonged exposure.** Representative DsRAN-NMF-enhanced line-scanning hyperspectral images show nanoplastic signals in the caudal vasculature over consecutive time points from 0 to 1.5 s. Nanoplastic particles are shown in green and vascular structures are shown in blue. The enlarged tail region highlights two distinct dynamic behaviors. In the orange dashed region, nanoplastic signals remain spatially persistent across consecutive frames, indicating local particle retention or aggregation within the distal vessel region. In contrast, the yellow dashed region shows a transient nanoplastic signal that disappears within the following time frame, consistent with rapid particle passage through the microvasculature. These observations demonstrate that DsRAN-NMF-enhanced hyperspectral imaging can distinguish relatively static vessel-associated nanoplastic accumulation from dynamic circulating particles in vivo.

**S18. Performance of DsRAN-NMF in human prostate tissue gland hyperspectral imaging.**

To evaluate the cross-domain versatility of DsRAN-NMF in biological hyperspectral imaging, we constructed few-shot training and validation sets using an in-house human prostate tissue hyperspectral dataset. Prostate gland tissue sections were stained with hematoxylin and eosin (H&E) and obtained from Carlina Biologicals. Hyperspectral images were acquired using a self-built line-scanning hyperspectral microscope configured with a 10× objective. For the training dataset, low signal-to-noise ratio (SNR) hyperspectral images were acquired under low illumination power (50 μW) and short exposure time (30 ms), denoted as Noisy images. In contrast, high SNR hyperspectral images were obtained under high illumination power (0.1 mW) and long exposure time (300 ms), referred to as High SNR reference (HIGH SNR REFERENCE) images. The data preprocessing method is the same as the main dataset, with 6 pairs used for training and 300 pairs used for testing.

**Fig. S16. Results of remote sensing HSI denoising using DsRAN-NMF on human prostate tissues gland datasets with 6 pairs training data (**The 20, 36, and 43 hyperspectral channels was set as RGB channels for visualization.)

DsRAN-NMF consistently demonstrated superior few-shot denoising performance, achieving significant improvements with only 6 training pairs: a +7.7 dB gain in PSNR, a 59.3% improvement in SSIM, and an 80% reduction in SAM. As shown in Fig. S16, noise was effectively removed while biologically relevant structures such as the fibromuscular stroma were clearly resolved and exhibited high structural consistency with the High SNR reference (HIGH SNR REFERENCE). Furthermore, the method accurately restored the spectral profiles that had been distorted by noise, successfully recovering subtle yet diagnostically important spectral differences between tissue types, such as those between prostatic acini/glands and the fibromuscular stroma, which were indistinguishable in the noisy input. These results confirm the capability of DsRAN-NMF in restoring and enhancing biological hyperspectral imagery with high spectral-spatial fidelity.

**S19. Performance of DsRAN-NMF in remote sensing, industrial inspection and unmanned aerial vehicle-based hyperspectral imaging.**

To evaluate the versatility of the DsRAN-NMF network across different hyperspectral domains, we assembled small-sample training and validation sets from three public benchmarks: Pavia (remote sensing)(1), ICVL (hyperspectral camera)(2) and SOC710-VP (hyperspectral camera)(3). DsRAN-NMF consistently demonstrated superior few-shot denoising performance across all domains, achieving significant improvements with just 12 training pairs:

(1). Pavia dataset: +14.0 dB PSNR gain, 83.5% reduction in SAM.

(2). ICVL dataset: +23.5 dB PSNR gain, 87.5% reduction in SAM.

(3). SOC710-VP dataset (real noise): +5.6 dB PSNR gain, 42.3% reduction in SAM.

Remarkably, this minimal training requirement enables rapid adaptation to novel hyperspectral applications - spanning remote sensing, macroscopic scenes (buildings/lawns), and microscopic still-life objects (vases/fruits) - while maintaining reconstruction fidelity.

(1) *Methods*

For Pavia, **12 noisy-clean image pairs** (Simulated noisy with 0.1 gauss noise) were used for training and 20 for validation with no overlap between the two sets; for ICVL, 12 noisy-clean (Simulated noisy with 0.3 gauss noise) image pairs were used for training and 13 pairs were allocated to validation likewise drawn from images disjoint from the training data. For SOC710-VP, 12 noisy-clean image pairs (realistic data) were used for training and 47 pairs for validation; The Pavia data, captured by the ROSIS-3 sensor over northern Italy, comprise two scenes-Pavia University (610 × 340 pixels, 103 bands) and Pavia Center (1096 × 715 pixels, 102 bands). The ICVL dataset, provided by Ben-Gurion University, contains natural scenes (1300 × 1392 pixels, 31 bands) acquired via a Specim PS Kappa DX4 camera mounted on a rotating stage. The SOC710-VP dataset was manufactured by Surface Optics Corporation (SOC), USA, using a SOC710-VP hyperspectral camera equipped with a silicon-based charge-coupled device (CCD) and an integrated scanning system, offering HSIs with 696 × 520 pixels in spatial resolution and 256 spectral bands from 376.76 nm to 1075.80 nm at 2.7 nm interval.

All images were preprocessed to satisfy deep-learning requirements. First, spectral dimensionality was standardized to 96 bands: the bands of ICVL and SOC710-VP were upsampled to 96 by linear interpolation, while bands of Pavia were truncated to the first 96. Next, for ICVL and Pavia datasets, gaussian noise of varying standard deviations was added into each image within its original dynamic range, followed by normalization. Finally, both clean and noisy images were cropped into 96 × 256 × 256-pixel patches and saved in uint16 format within a structured directory hierarchy.

(2) *Results*

Using just 12 paired training samples corrupted with Gaussian noise (σ = 0.1) and 300 training epochs, the DsRAN-NMF network achieves high-performance denoising on the Pavia hyperspectral dataset (Fig. S9). The PSNR increases from 25.76 ± 0.06 dB to 39.32 ± 0.19 dB, while the mean SAM decreases sharply from 0.473 ± 0.003 to 0.078 ± 0.002. Spatially, DsRAN-NMF elegantly reconstructs noise-obscured structures-such as building façades-with near-pixel-level fidelity to the High SNR reference. Spectrally, it preserves material signatures with minimal angular distortion, confirming its efficacy in both domains.

**Fig. S17. Results of remote sensing HSI denoising using DsRAN-NMF on Pavia datasets with 12 pairs training data (**The 25, 52, and 86 hyperspectral channels was set as RGB channels for visualization.)

**Fig. S18. Results of hyperspectral camera images (architecture and grassland) denoising using DsRAN-NMF on ICVL datasets with 12 pairs training data. (**The 25, 53, and 86 hyperspectral channels was set as RGB channels for visualization.).

On the ICVL ground‐based hyperspectral dataset, DsRAN‐NMF achieves similarly outstanding results: using only 12 training samples, delivering a PSNR gain of ~23.5 dB, an SSIM increase of ~44.7%, and an 87.5% reduction in SAM (Fig. S10). Qualitative evaluation confirms that the network reliably reconstructs both architectural and vegetated surfaces from extreme noise corruption, yielding outputs that are virtually indistinguishable from the High SNR reference and free of artifacts or hallucinations. Notably, all twelve training pairs depict building scenes-none resemble the grass‐and‐signpost example-yet the model faithfully denoises this novel vegetation scenario (Fig. S18(a)). This performance demonstrates that DsRAN‐NMF learns a content‐agnostic representation that synergistically combines spectral and spatial priors, thereby exhibiting strong generalization across unseen domains.

**Fig. S19. Results of hyperspectral camera images (architecture and** **close still life) denoising using DsRAN-NMF on SOC710-VP datasets with 12 pairs training data. (**The 15, 43, and 96 hyperspectral channels was set as RGB channels for visualization.).

DsRAN-NMF also demonstrates excellent denoising performance on the real-noise SOC710-VP dataset, achieving improvements of ~5.6 dB in PSNR and a reduction of 42.3% in SAM (Fig. S19). Visual inspection confirms that DsRAN-NMF effectively restores noise-obscured edges of the orange and faithfully reproduces color transitions. These results demonstrate significant quality enhancement in the hyperspectral images (HSI) from both spectral and spatial perspectives. The performance on SOC710-VP further indicates DsRAN-NMF's capability for high-quality restoration of everyday objects.

Furthermore, DsRAN-NMF delivers robust reconstruction across diverse domains, including remote sensing, distant-view, and close-range hyperspectral scenes. Remarkably, effective pre-training for denoising requires only 12 paired training samples. This minimal data requirement, combined with its broad content adaptability, positions DsRAN-NMF as a highly promising solution for hyperspectral image denoising across various application fields.

**S20. Zebrafish uptake nanoplastics experiment.**

Zebrafish embryos were purchased from the National Aquatic Biological Resource Center (China) and incubated in a 26 °C incubator after purchase. All animal procedures were complied with guidelines of Chinese Council on Laboratory Animal Care, and the fish were carefully treated. In this study, zebrafish were used both in training data collection and zebrafish uptake nanoplastics experiments.

(1) zebrafish-nanoplastics coexist sample for training datasets collection

To generate co-localized zebrafish and nanoplastic samples, 5-7-day post-fertilization (d.p.f). Tg(s843) zebrafish were euthanized in 2000 mg/L tricaine (MS-222), then fixed in 4% paraformaldehyde for 20 mins. After PBS washes, each fish was embedded in 1.5% (w/w) agarose containing 0.2 µm yellow-green fluorescent nanospheres (FluoSpheres, Thermo Fisher, 2% solids). Samples were cleared with UbasM2 solution for 20 mins and then stored at 4°C until hyperspectral imaging.

(2) Zebrafish uptake nanoplastics experiment

**Fig.S20. Experimental timeline for nanoplastic exposure and hyperspectral observation in zebrafish larvae**

Prior to exposure, zebrafish eggs were incubated in ultrapure water at 28°C until 5 d.p.f. 40 mL of nanoplastic exposure solution was prepared at a concentration of 5 mg/L in ultrapure water, into which the zebrafish larvae were exposed. After 24 hours of exposure, the fish was transferred to clean ultrapure water (nanoplastic-free), followed by 80% water renewal every 24 hours. Individual larvae were sampled for observation at designated time points (12 h, 24 h, 36 h, 72 h).

For HSPEC-LSFM imaging, zebrafish were anesthetized in 50 mg/L MS-222 solution and then carefully transferred into a cylindrical mold containing approximately 37°C, 1.5% low-melting-point agarose. Prior to agarose solidification, the fish orientation was gently adjusted to a specific position (e.g., lateral recumbency) for optimal observation. Following complete solidification, the agarose column was vertically mounted on the sample holder of the HSPEC-LSFM system. Z-stack images were then acquired to analyze the spatial distribution and accumulation of microplastics within the body.

For 2D line-scanning hyperspectral imaging, zebrafish were similarly anesthetized (50 mg/L MS-222) and then carefully transferred into a glass-bottom dish containing approximately 37°C, 1.5% low-melting-point agarose. After agarose solidification immobilized the specimen, the dish was placed directly onto the stage of the 2D line-scanning hyperspectral microscopy for time-lapse imaging.

**S21. Characterization results of the developed imaging platform based on HSPEC-LSFM.**

The fluorescent bead phantom consisting of a low concentration suspension of fluorescent beads (FluoSpheres™ Aldehyde-Sulfate Microspheres, 1.0 µm, yellow-green fluorescent, 505/515, 2% solids) embraced in 1.5% agarose (A7002, SLBK5425V, Sigma Aldrich, St. Louis, MO, USA) were used to characterize the spatial resolution of HSPEC-LSFM, exhibiting a lateral resolution of 2.77 ± 0.29/3.31 ± 0.36 μm (direction of line-scanning and direction perpendicular to line scanning) and an axial resolution of 4.84 ± 0.59 μm (as shown in Fig. S21). The imaging exhibits uniform clarity across the field of view, with negligible chromatic aberration.

**Fig. S21. Spatial resolution characterization of HSPEC-FLSM using the fluorescent bead phantom including 3D intensity image and the intensity distributions along the x, y and z axis of beads with Gauss fitting line.**

The spectral resolution of the system was characterized using a standard mercury lamp source (HG-CAL, ZCN, China). Figure S13 displays the spectral curve acquired within the 400-700 nm range using the Princeton Instruments LightField spectroscopy software, showing characteristic mercury emission peaks at 436.23 nm, 546.34 nm, 577.26 nm, and 579.43 nm, consistent with the reference spectrum. Since mercury lamps provide discrete emission lines with theoretical linewidths negligible compared to the hyperspectral instrument's resolution limit, the spectral resolution was determined directly as the measured full width at half maximum (FWHM) of the acquired peaks, yielding a value of 0.91 ± 0.02 nm.

**Fig. S14. Spectral curve of standard mercury lamp source captured by HSPEC-LSFM with Lightfield software.**

**S22. i*n vivo* HSPEC-LSFM imaging of zebrafish with sustained exposure to nanoplastics - 48 hours and 72 hours.**

Separately from the pulsed-exposure design in the main manuscript, nanoplastic migration was evaluated following a continuous exposure protocol. This procedure subjected zebrafish larvae to uninterrupted immersion in the specified nanoplastic suspension. Prior to exposure, fertilized eggs were incubated in 28°C ultrapure water until 5 days post-fertilization (d.p.f.). Larvae were then exposed to 40 mL of 5 mg/L nanoplastic suspension prepared in ultrapure water. To maintain constant exposure concentrations, 80% of the solution was renewed every 24 hours. Individual larvae were sampled for analysis at designated intervals (12-, 24-, 36-, and 72-hours post-exposure initiation).

**Fig. S22. 3D visualizations via MIP rendering and the corresponding segmentation results of zebrafish embryo enhanced by the low-data pre-trained DsRAN-NMF network at 36 hours (a) and 72 hours (b)of sustained nanoplastics exposure. (c) 2D line-scanning hyperspectral images of zebrafish at 72 hours of sustained NPs exposure.**

The migration of nanoplastics from the surrounding medium into the organism occurs as a sustained process, upon continuous exposure of zebrafish to a NPs-containing environment. Throughout the experimental period, NPs signals were observed in the intestinal tract of zebrafish, suggesting that the gastrointestinal route may serve as the primary pathway for NPs entry into the vascular system under continuous exposure conditions. Similar to the results observed under "pulsed exposure" in the main text, a substantial accumulation of NPs was detected in the dorsal aorta of the zebrafish abdomen at 36 h of continuous exposure, while no significant signals were present in secondary vessels at that time point (Fig. S22(a)). By 72 h of continuous exposure, widespread NPs accumulation was evident throughout various regions of the vascular system (Fig. S22(b)). Hyperspectral unmixing of NPs and vascular signals further confirmed that large aggregates of NPs were present in the intersegmental vessels. Notably, NP signals were more frequently observed at vascular bifurcations and curvature sites, which may be attributed to localized flow disturbances and lower shear stress that favor particle deposition. This observation is consistent with findings from the "pulsed exposure" condition, indicating that NPs enter the vascular system via the intestine and subsequently translocate to progressively smaller vessels.

Beyond vascular translocation via the intestines, nanoplastics underwent predominant digestive clearance. At the 72h timepoint, we directly captured the expulsion of significant nanoplastic aggregates through fecal matter during defecation events (Fig. S22(c)).

**S23. Image thresholding segmentation and visualization processing.**

Image segmentation, geometric modeling, and quantitative analysis were performed on the NMF‐decomposed three‐dimensional volumes of nanoplastic particles and the vascular network. First, a binary mask of the vasculature was extracted via intensity thresholding, and a triangular surface mesh was generated to represent its 3D geometry. This mesh was rendered as a semi‐opaque green volume. An identical procedure was then applied to the nanoplastic channel, after which the nanoplastic volume was spatially overlaid onto the segmented vascular model, enabling direct localization of nanoplastics against the anatomical backdrop of the blood vessels. Overlap between the nanoplastic and vascular meshes was computed at the voxel level to determine which particles resided within the vessel interior. From these data, the total vessel volume, total nanoplastic volume, and intravascular nanoplastic volume were quantified.

**Fig. S23 Segmentation visualization of ROI from Fig. 5 (a), Fig. S12, and Fig. S13.**

For visualization, a fixed light source-stationed at the camera position-was introduced using a Gouraud shading model to accentuate volumetric depth and surface detail. All axes, tick marks, and grid lines were hidden, and the viewing bounds were set to match the spatial dimensions of the vascular segmentation so that both the vessel model and nanoplastic volume shared a common coordinate system. A full‐HD off‐screen rendering window was created to capture high‐resolution axial slices. During slice‐by‐slice traversal, the camera was switched to an orthographic top‐down view, flattening the selected layer’s vasculature and nanoplastic volumes into a 2D plane. The depth range was clamped to the thickness of each slice, and all nonessential visual elements remained suppressed to preserve rendering clarity. After each frame was rendered, the image buffer was exported as an RGB array; pure white pixels (representing empty background) were set to transparent to preserve only the biological structures. Each processed slice was written in lossless format to a multi‐page TIFF file: the first slice created (or overwrote) the file, and subsequent slices were appended in sequence. The resulting TIFF stack faithfully depicts the spatial relationship between the vascular network and nanoplastic distribution, facilitating both comprehensive 3D volumetric analysis and detailed layer‐by‐layer inspection.

Fig. S23 displays the threshold-segmentation results for the ROIs in Fig. 6(a), and Fig. S22 at 12 h, 48 h, and 72 h. Following segmentation and 3D rendering, the spatial progression of nanoplastic distribution is evident: at 12 h, particles remain external to the vasculature, confined primarily to the gills and head surface; by 48 h, a large accumulation appears within the dorsal aorta; and at 72 h, nanoplastics are dispersed throughout the body, including secondary vessels in the tail.

**S24. Quantitative metrics used for model evaluation.**

For denoised hyperspectral data, quantitative evaluation must address both spatial fidelity and spectral integrity across hundreds of contiguous wavelenHigh SNR referenceh bands. Peak Signal-to-Noise Ratio (PSNR) provides a fundamental measure of overall reconstruction accuracy by computing the logarithmic ratio between the maximum possible pixel value and the mean-squared error (MSE) between the denoised and reference images:

$$PSNR=10\cdot\log(\frac{MAX_{I}^{2}}{MSE})$$

$$MSE=\frac{1}{mn}\sum_{i=0}^{m-1} \sum_{j=0}^{n-1} ||I(i,j)-K(i,j)||^{2}$$

Although widely used for image restoration, PSNR captures only pixel‐wise intensity differences and does not directly reflect perceptual or spectral distortions in hyperspectral data. To complement PSNR, the Structural Similarity Index (SSIM) quantifies the preservation of local texture and edge information by comparing luminance, contrast, and structural components within small windows, thereby offering insight into how well denoising maintains the spatial features that are critical for downstream tasks such as segmentation or object detection:

$$SSIM(\text{x},y)=\frac{(2\mu_{x}\mu_{y}+C_{1})(2\sigma_{xy}+C_{2})}{(\mu_{x}^{2}+\mu_{y}^{2}+C_{1})(\sigma_{x}^{2}+\sigma_{y}^{2}+C_{2})}$$

Where x, y the images to be compared. μx, μy the mean of the images. σx2, σy2 the variance of the images, σxy the covariance of image x and y. C1 =(K1L)2, C2 = (K2L)2 the small constants to avoid zero denominator, where L is the dynamic range of images (e.g. 65535 for 16-bit images).

However, both PSNR and SSIM treat each spectral band independently, ignoring inter‐band relationships. The Spectral Angle Mapper (SAM) metric addresses this limitation by measuring the spectral angle between each pixel’s high‐dimensional reflectance vector in the denoised data and the corresponding vector in the High SNR reference; because SAM is invariant to scalar multiplicative changes, it effectively captures distortion in spectral shape and is particularly sensitive to deviations in material signatures:

$$SAM(x,y)=\arccos(\frac{\langle x,y\rangle}{||x||\cdot||y||})$$

Where x, y the spectral vectors to be compared, B is the number of bands.

Finally, Spectral Information Divergence (SID) evaluates the divergence between the probability distributions of spectral vectors by treating each band’s reflectance as a normalized probability, thereby quantifying the discrepancy in statistical spectral information. A low SID indicates that the denoised pixel retains a distribution of intensities across wavelenHigh SNR referencehs that closely matches the original.

$$SID(x,y)=D(x||y)+D(y||x)$$

$$D(x||y)=\sum_{i=1}^{B} x_{i}\log(\frac{x_{i}}{y_{i}})$$

Where x, y the sum-to-1 normalized spectral vector, B the number of bands. D the Kullback Leibler divergence.

By jointly reporting PSNR, SSIM, SAM, and SID, this study ensures a holistic assessment of denoising performance: PSNR and SSIM verify spatial reconstruction quality, while SAM and SID confirm that subtle spectral features and material‐specific signatures remain intact after noise reduction.

**S25. Low-data learning HSI denoising results using SOTA deep-learning methods.**

Current SOTA fully supervised networks exhibit significant sensitivity to training dataset size and struggle with data reduction. As shown in Fig. S24 (i.e. the supplementary of Figure 5), when the training set size decreased from 100% (220 pairs) to 25% (55 pairs), these SOTA networks suffered a pronounced performance deterioration: DSTrans and HSI-DeNet displayed severe overfitting artifacts (ghosting), while QRNN3D exhibited spectral reconstruction errors, evident as color shifts in both blood vessels and nanoplastics. In contrast, DsRAN-NMF maintained robust image quality and factual consistency under these conditions.

**Fig. S24. 3D visualizations via MIP rendering of zebrafish denoised using learning-based methods with full-data learning and low-data learning (25% datasets) using DsRAN-NMF network and other SOTA methods respectively.**

This pronounced performance degradation underscores a fundamental limitation of fully supervised paradigms: their empirical risk minimization frameworks intrinsically demand large, representative datasets to generalize effectively. When training data becomes scarce (<100 pairs), such networks fail to adequately constrain their high-capacity architectures-typically comprising millions of parameters. Consequently, they may resort to memorizing noise patterns rather than learning physically meaningful features, resulting ghosting artifacts and manifest spatial hallucination from undersampled feature learning of DSTrans, QRNN3D and HSI-DeNet. These failure modes collectively reveal that without explicit physical priors, SOTA networks become critically dependent on exhaustive data coverage to approximate the hyperspectral manifold.

**S26. DsRAN-NMF at varying training data levels.**

To systematically evaluate the dependence of DsRAN-NMF on training-set size, restoration performance was first examined using the full dataset of 220 paired images and progressively reduced subsets containing 55, 26, and 13 pairs. Representative restoration results are shown in Fig. S25(a), and the enlarged ROI and corresponding spectral profiles are shown in Fig. S25(b). The principal nanoplastic-associated structures and characteristic spectral profiles remained broadly consistent as the training dataset was reduced from 220 to 13 pairs.

To further evaluate the effective restoration performance, quantitative metrics were calculated using 55, 26, 13, 9, 6, and 4 paired training images on the same independent test set. As shown in Fig. S25(c), SSIM remained relatively stable and SAM remained low when the number of training pairs was reduced from 55 to 6, although metric variability gradually increased at smaller dataset sizes. Models trained with 6-13 pairs continued to provide substantial improvements in structural and spectral fidelity relative to the noisy input. However, when the training set was reduced to 4 pairs, SSIM decreased markedly and SAM increased, accompanied by visible structural and spectral instability in the restored result (Fig. S25(d)). These observations show that approximately 6-13 paired images can be used for the present zebrafish fHSI dataset.

**Figure S25. Effect of training-set size on DsRAN-NMF restoration performance.** (a) Representative restored hyperspectral images obtained using the full training dataset of 220 pairs and reduced datasets of 55, 26, and 13 pairs, together with the noisy input and high-SNR reference. (b) Enlarged ROI and corresponding normalized spectral profiles for the noisy input, high-SNR reference, and models trained with 220, 55, 26, and 13 paired images. (c) SSIM and SAM as functions of training-set size using 55, 26, 13, 9, 6, and 4 paired images. Restoration performance remained relatively stable from 55 to 6 training pairs but deteriorated markedly when the dataset was reduced to 4 pairs. Error bars indicate variation across the evaluated test images. (d) Representative restoration result obtained using 4 training pairs, showing visible structural and spectral instability under extremely limited training data.

This data efficiency is consistent with the low-dimensional structure of fluorescence hyperspectral data. Although each HSI contains many spectral channels, the dominant spectral variation in the present task is primarily governed by a limited number of fluorescent contributors and their mixtures. The NMF-guided spectral constraint allows the restored spectra to remain consistent with this compact endmember-mixing manifold rather than treating each spectral channel as an independent prediction target. Therefore, a representative low-data subset that captures the major spatial structures, spectral components, signal levels, and degradation patterns may contain sufficient information for learning the dominant spectral manifold. This is also supported by the effective low-data results obtained in other hyperspectral domains, summarized in Table S7.

**Table S7. Training-set sizes used for low-data DsRAN-NMF restoration across different hyperspectral domains.**

| **Dataset/domain** | **Imaging content** | **Noise/reference setting** | **Training pairs** | **Test/validation pairs** |
| --- | --- | --- | --- | --- |
| Zebrafish-nanoplastic fHSI | Fluorescent vasculature, nanoplastics and autofluorescence | Experimental low-/high-SNR pairs | 6-13 | 22 |
| Human prostate tissue fHSI | H&E-stained prostate tissue | Experimental low-/high-SNR pairs | 6 | 300 |
| Pavia | Remote-sensing scenes | Simulated Gaussian noise | 12 | 20 |
| ICVL | Natural and architectural scenes | Simulated Gaussian noise | 12 | 13 |
| SOC710-VP | Macroscopic objects and scenes | Real-noise paired data | 12 | 47 |

It is noted that the 6-13-pair range here is specific to the datasets and experimental conditions evaluated here. The number of paired samples required for reliable restoration may vary with the intrinsic dimensionality and complexity of the spectral manifold, endmember variability, spatial heterogeneity, noise characteristics, imaging configuration, and domain shift. In general, the selected training set must contain sufficient and representative information to capture the principal spectral manifold and the corresponding degradation process.

**Reference**

1.	M. V. M Graña, B Ayerdi. (<https://www.ehu.eus/ccwintco/index.php/Hyperspectral_Remote_Sensing_Scenes>).

2.	B. Arad, O. Ben-Shahar, in *Computer Vision - ECCV 2016,* B. Leibe, J. Matas, N. Sebe, M. Welling, Eds. (Springer International Publishing, Cham, 2016), pp. 19-34.

3.	T. Zhang, Y. Fu, J. Zhang, Guided Hyperspectral Image Denoising with Realistic Data. *International Journal of Computer Vision* **130**, 2885-2901 (2022).
